# Regulated Cell Death in Fungi, the Role of Metacaspases and Assay Techniques

**DOI:** 10.3390/mps9030083

**Published:** 2026-05-31

**Authors:** Derek Wilkinson

**Affiliations:** BIOCEV, Faculty of Science, Charles University, 128 00 Prague, Czech Republic; derek.wilkinson@natur.cuni.cz; Tel.: +420-325873934

**Keywords:** apoptosis, PCD, RCD, caspase, metacaspase, fungi

## Abstract

Caspases orchestrate metazoan apoptosis, regulating processes such as embryogenesis, the death of old and infected cells and immune tolerance. Structural orthologs of caspases have been identified in bacteria, plants, protists and fungi and regulated cell death has been demonstrated in these organisms. This led some researchers to conclude that fungal metacaspases might perform a similar function to caspases. This review discusses regulated cell death, beginning with an account of RCD and the central role of caspases in mammalian RCD. It goes on to give examples of RCD in fungi, compares the structure and activity of caspase orthologs and outlines examples of metacaspase-dependent and metacaspase-independent cell death in fungi, focusing on *S. cerevisiae*. Finally, it addresses the question “are metacaspases caspases?”, identifies alternative cell death proteases and recommends future research objectives.

## 1. Introduction

Apoptosis, a form of regulated cell death (RCD), is vital for mammalian development, positive and negative selection of immune cells and turnover of old and worn-out cells [1,2,3,4,5,6,7,8,9,10,11,12,13,14,15,16]. Apoptosis of superfluous cells sculpts precise shapes, such as fingers and toes, organs and tissues. B- and T-lymphocytes that do not produce operational antigen receptors are removed via apoptosis. Weak interactions of T-cells with the major histocompatibility complex and strong reactions to self-antigens trigger apoptosis. B-cells are also subject to negative selection. Exactly 131 *Caenorhabditis elegans* somatic cells undergo apoptosis during development. Balanced mitosis and apoptosis maintain cell numbers and dysfunctional apoptosis can lead to cancer or inflammation.

The discovery of structural caspase orthologs [17] and apoptosis-like cell death in fungi, protists, plants, etc., suggested that apoptosis is conserved throughout the tree of life [18,19,20,21,22]. Caspase orthologs were also identified in cyanobacteria, brown algae, proteobacteria, archaea, actinobacteria and viruses [23,24,25,26,27]. RCD may be apoptotic, autophagic or necrotic (reviewed by [28]).

This review describes mammalian apoptosis, regulation by caspases and techniques for detecting RCD characteristics. It also discusses RCD and caspase orthologs in other organisms, focusing mainly on fungi. Methods for detecting fungal RCD are considered, as is the great debate: are metacaspases caspases?

## 2. Characterization of Mammalian Apoptosis

### 2.1. Physiological Changes During Apoptosis

**Figure 1:**
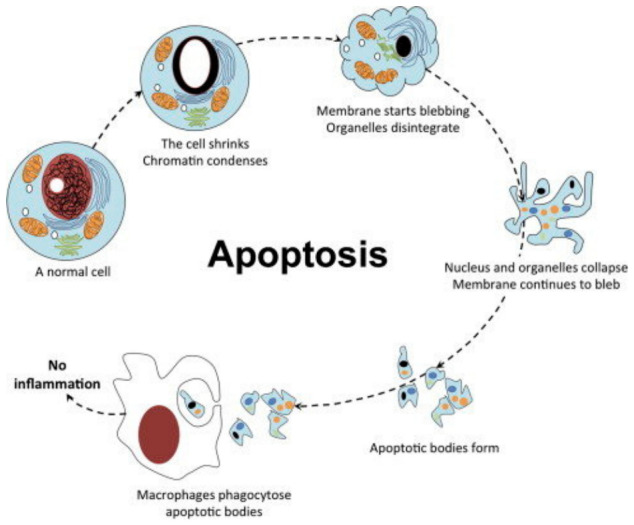
Hallmarks of mammalian apoptosis. When mammalian cells undergo apoptosis, the cells shrink, and chromatin condenses during the initial stages. Later, parts of the plasma membrane swell, forming bubbles (blebs). Then, organelles, such as the nucleus and mitochondria, disintegrate. Finally, cell contents separate into membrane-bound “apoptotic bodies”. These are removed by phagocytes, ensuring that potentially toxic substances are safely disposed of and preventing widespread inflammation. Reproduced from [32]. © 2015 Abou-Ghali and Stiban. CC BY-NC-ND License (http://creativecommons.org/licenses/by-nc-nd/4.0/, Accessed on 27 May 2026).

During apoptosis, mammalian cells (Figure 1) undergo blebbing of the plasma membrane (PM), flipping of phosphatidylserine (PS) to the outer PM layer, reduced mitochondrial membrane potential, cell shrinkage, chromatin condensation, DNA fragmentation and disintegration of the cell into apoptotic bodies [29,30,31,32] that are cleared by phagocytes so that cell contents do not damage surrounding tissues or trigger inflammatory responses [33,34,35]. Apoptosis is ordered cell death, regulated by genetically encoded proteins and conserved pathways, and necrosis (except programmed necrosis) is accidental cell death [36,37]. Necrosis involves swelling of cells and organelles, PM disruption and no DNA fragmentation.

### 2.2. Detecting Apoptosis

**Figure 2:**
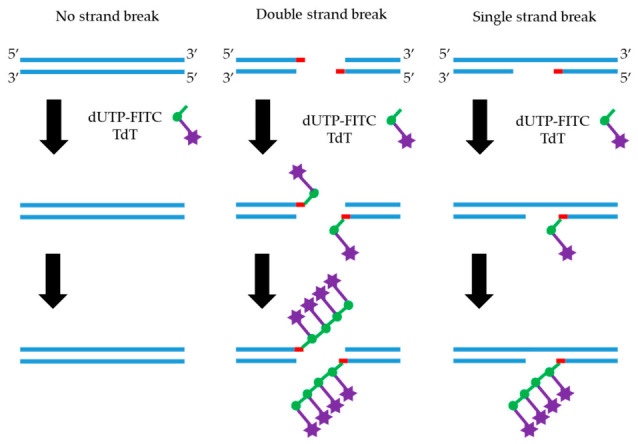
TUNEL staining. During apoptosis, the DNA strand breaks expose 3′ end hydroxyl (-OH) groups (red lines). Terminal deoxynucleotidyl transferase (TdT) labels 3′-OH with dUTP (green circles) conjugated with a fluorescent moiety such as FITC (purple stars) and leads to the formation of chains of FITC-labeled dUTPs. An alternative is to use an antibody raised against dUTP and conjugated with a fluorescent moiety. Adapted from [43] with permission from Elsevier. Arrows represent addition of labeled dUTPs.

Cell shrinkage, membrane blebbing, chromatin condensation and the formation of apoptotic bodies may be observed using light or electron microscopy and chromatin condensation by fluorescence microscopy and DNA staining with 4,6-diamidino-2-phenylindole-dihydrochloride (DAPI) or Hoechst stain [38,39,40,41,42]. Prior staining with eosin-methylene blue helps to distinguish the cytoplasm and nucleus and DNA fragmentation may be studied using flow cytometry or gel electrophoresis. Flipping of PS to the outer PM leaflet is visualized using annexin V, which binds strongly to PS. The DNA strand breaks expose 3′ end hydroxyl groups, which can be visualized via dUTP Nick End Labeling (TUNEL) staining [43]. Terminal deoxynucleotidyl transferase (TdT) attaches 5-bromo-2′-deoxyuridine 5′-triphosphate (Br-dUTP) to 3′ end hydroxyl groups. Either the dUTP is directly labeled with a fluorescent moiety or a FITC-labeled antibody. Ab-FITC is bound to Br-dU (Figure 2) and the fluorescein isothiocyanate (FITC) detected using fluorescent microscopy.

### 2.3. Mammalian Caspases

**Figure 3:**
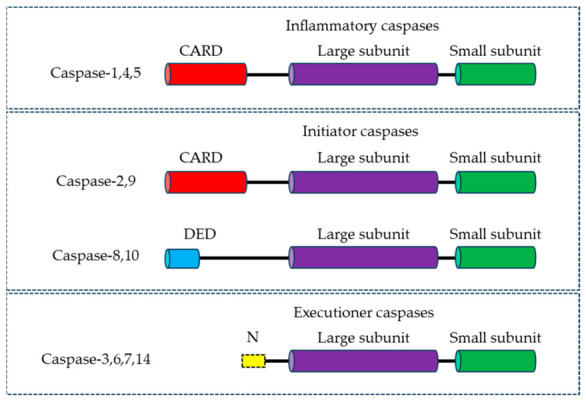
Structure of human caspases. Caspases-1, -4 and -5 are inflammatory caspases with roles in the processing of immune mediators such as interleukin-1β. Caspases-2, -8, -9 and -10 are initiator caspases that are activated by extrinsic signals and, in turn, activate executioner caspases. Caspases-3, -6 and -7 are executioner caspases that cleave target proteins and peptides, leading to apoptosis via gain or loss of function of the targets. Caspases-1, -2, -4, -5 and-9 include caspase recruitment domains (CARDs) while caspases-8 and -10 include death effector domains (DEDs) and both CARDs and DEDs mediate protein interaction. Executioner caspases may or may not have small N-terminal domains (N). All caspases include a small and large catalytic subunit. Adapted from [44] with permission from Cold Spring Harbor Laboratory Press.

**Figure 4:**
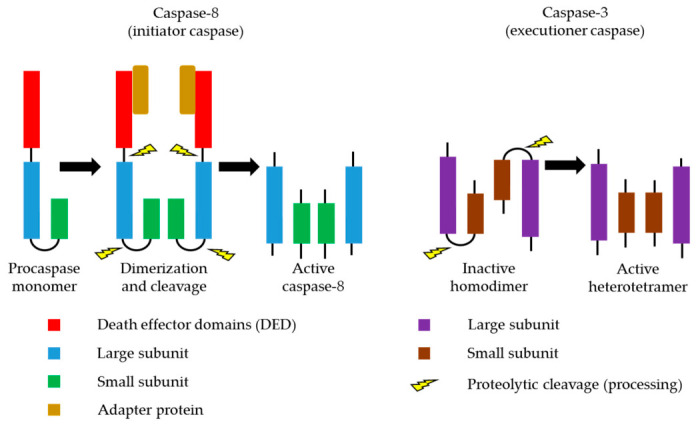
Activation of initiator and executioner caspases. Procaspase-8 is cleaved between the large and small subunit and between the large subunit and pro-domain, forming active caspase-8. Caspase-3 forms dimers and cleavage between the large and small subunits forms active caspases. Adapted from [47] with permission from Springer Nature.

Apoptosis is orchestrated by caspases, i.e., cysteine-dependent, aspartyl proteases, which cleave proteins after aspartate and have a conserved cysteine/histidine catalytic dyad [44,45]. Proteolysis is activated via cleavage and association of the large and small subunits. Some caspases regulate inflammation and others regulate apoptosis. The former group includes caspase-1, -4 and -5, which cleave proproteins to yield mature inflammatory mediators such as interleukin-1β. The latter group includes initiator and executioner caspases (Figure 3). Initiator caspases are activated by intrinsic or extrinsic signaling via interaction between DED and CARD domains, and initiator caspases activate executioner caspases, which cleave downstream proteins, leading to apoptosis. Caspase-8 associates with adapter proteins (Figure 4), bringing two proteins closer and activating proteolysis [46,47]. Two large subunits and two small subunits form the active caspase-8 heterotetramer. Caspase-3 forms dimers with one monomer inverted with respect to the other. Activated initiator caspases activate caspase-3 via cleavage between the large and small subunits.

### 2.4. Apoptosis Pathways

**Figure 5:**
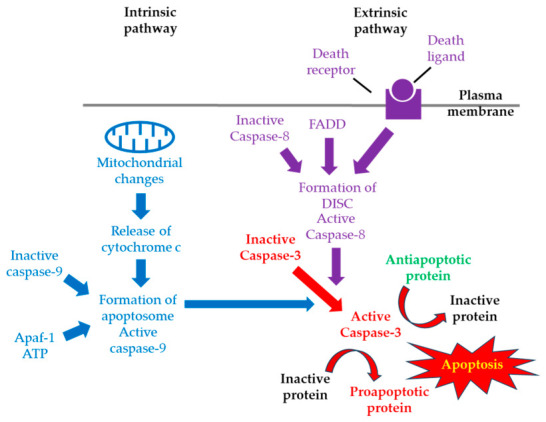
Intrinsic and extrinsic pathways. Intrinsic and extrinsic apoptosis converge on executioner caspases, such as caspase-3, which cleaves target proteins, activating some and inactivating others, triggering apoptosis. Adapted from [49].

Apoptosis is triggered via different pathways [15,48,49]. The intrinsic (mitochondrial) pathway occurs in response to, e.g., lack of oxygen (Figure 5) via altered mitochondrial physiology, loss of membrane potential and cytochrome c release. Cytochrome c, caspase-9 and Apaf-1 form the apoptosome and activate caspase-9. Extracellular signaling activates the extrinsic (death receptor) pathway via binding of ligands (e.g., tumor necrosis factor alpha—TNFα) to surface receptors. Adapter proteins (e.g., FADD) and caspase-8 form the death-inducing signaling complex (DISC) and activate caspase-8.

### 2.5. Extrinsic Apoptosis and the DISC

**Figure 6:**
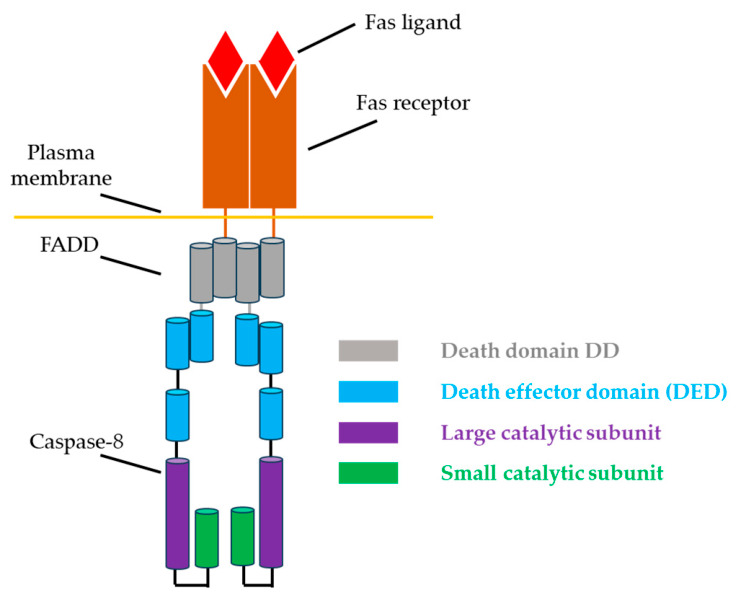
Death-inducing signaling complex. The DISC mediates extrinsic apoptosis and is formed when death ligands bind to death receptors at the cell surface, causing the death receptors to cluster together. Death receptors associate with the adapter protein FADD via their death domains and FADD with caspase-8 (or caspase-10) via death effector domains. Clustering of death receptors brings two caspase proteins close together and they form an active heterotetramer. Adapted from [52] with permission from Springer Nature.

Death ligands induce apoptosis by attaching to death receptors [48,50,51,52]. For example, tumor necrosis factor-α (TNF-α) binds to tumor necrosis factor receptor 1 (TNFR1). When FasL binds to Fas (Figure 6), the receptors cluster, promoting interaction with the Fas-associated protein with death domain (FADD). The latter recruits procaspase-8 via death effector domains (DEDs), forming the death-induced signaling complex (DISC) (Figure 6). This promotes cleavage of the procaspase and formation of a heterotetramer (active caspase-8), which cleaves and activates caspase-3.

### 2.6. Intrinsic Apoptosis and the Apoptosome

**Figure 7:**
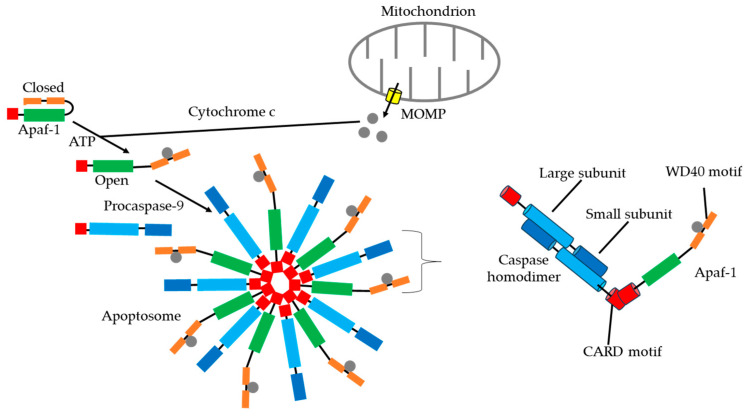
Apoptosome. Once pro-apoptotic factors trigger the permeabilization of the mitochondrial membrane, cytochrome c is released from the mitochondria, and in the presence of ATP, associates with Apaf-1 and procaspase-9 to form the apoptosome. The latter promotes apoptosis via proximity-dependent activation of caspase-9 by bringing two caspase molecules together to form a homodimer, thus forming an active caspase catalytic site. The bird’s eye view on the left shows caspase and Apaf-1 in the same plane but one extends above the plane and one below, as seen in the side view of one unit on the right. Caspase and Apaf-1 interact via their CARD motifs and Apaf-1 interacts with cytochrome c via its WD40 domains. Orange—WD40 motif; dark blue—small subunit; light blue—large subunit; red—caspase recruitment domain (CARD); MOMP—mitochondrial outer membrane permeabilization; Apaf-1—apoptotic protease-activating factor 1. Adapted from [54] with permission from Springer Nature.

Stimuli, such as changes in metabolism or the cell cycle, DNA damage, growth factor withdrawal, some drugs, lack of oxygen, heat shock, radiation and viruses activate the intrinsic apoptosis pathway (Figure 7) by inducing changes in mitochondria that lead to mitochondrial outer membrane permeability (MOMP) and the release of cytochrome c from the mitochondria [53,54].

### 2.7. Other Forms of Cell Death in Mammals

In 2023, the Nomenclature Committee on Cell Death (NCCD) recognized several forms of mammalian cell death in addition to apoptosis—pyroptosis, ferroptosis and necroptosis (the major three, which are all forms of regulated necrosis), and autosis, parthanatos, entotic cell death, NETotic cell death, lysosome-dependent cell death, mitochondrial permeability transition-driven necrosis, autophagy-dependent cell death, alkaliptosis, cuproptosis and PANoptosis [55]. The NCCD define these forms of cell death as follows:

#### 2.7.1. Pyroptosis

Pyroptosis involves the cleavage of gasdermin between the pore-forming N-terminus and the inhibitory C-terminus [56]. Caspases-1, -3, -4, -5, -8 and -11 can each cleave gasdermin D or gasdermin E, leading to the formation of pores in the plasma membrane, the movement of ions and water across the membrane and cell death. NLRP3 inflammasome-driven pyroptosis, involves the activation of pro-caspase-1 in the NLRP3, leading to cleavage of gasdermin, pore formation and cell death.

#### 2.7.2. Ferroptosis

Ferroptosis involves the peroxidation of fatty acyl groups of cellular lipids by iron-dependent ROS, leading to damage of the plasma membrane and of organellar membranes and thus cell death [57].

#### 2.7.3. Necroptosis

During necroptosis, the stress-dependent binding of a ligand to a receptor, such as tumor necrosis factor alpha (TNF-α) to TNFR, may lead to cell death via activation of the necrosome—a protein complex consisting of mixed lineage kinase domain-like protein (MLKL), receptor-interacting protein kinase 1 (RIPK1), and RIPK3 [58]. Successive phosphorylation leads to activation of MLKL, which attacks the plasma membrane.

#### 2.7.4. Autosis

Autosis may be triggered by extended autophagy in starving cells and involves specific changes in the physiology of various organelles, including the nucleus and ultimately loss of autophagosomes, autolysosomes and endoplasmic reticulum [59].

#### 2.7.5. Parthanatos

Parthantos involves hyperactivation of poly (ADP-ribose) polymerase 1, loss of Aif1 from the mitochondria and DNA destruction [60].

#### 2.7.6. Entosis

Entosis is the engulfment and digestion of one cell by another and involves Rho signaling and changes in the cytoskeleton of the engulfed cell and autophagosome involvement in digestion by the engulfing cell [61].

#### 2.7.7. NETosis

One of the ways in which neutrophils kill microorganisms is to release neutrophil extracellular traps (NETs)—sticky chromatin with attached granules containing hydrolytic enzymes, antimicrobial peptides, etc., and NET release leads to NETosis—regulating death of the neutrophil [62].

#### 2.7.8. Lysosome-Dependent Cell Death (LDCD)

LDCD involves changes in the permeability of lysosomes and release of lysosomal enzymes that mediate cell damage and death [63].

#### 2.7.9. MPT-Driven Cell Necrosis

Mitochondrial permeability transition-driven necrosis involves mitochondrial damage, increased permeability, loss of membrane potential and necrosis [64].

#### 2.7.10. Autophagy-Dependent Cell Death

Macroautophagy is important for recycling cellular material but can lead to autophagy-dependent cell death via excessive buildup of vacuoles [65].

#### 2.7.11. Alkaliptosis

In alkaliptosis, increased pH triggers cell death via NF-кB and STAT3 signaling [66].

#### 2.7.12. Cuproptosis [67]

Cuproptosis involves copper accumulation in cells due to dysfunctional copper homeostasis, leading to cell death via damage to iron–sulfur clusters of key enzymes and disruption of pyruvate dehydrogenase activity or ubiquitin-dependent protein degradation [68].

#### 2.7.13. PANoptosis

PANoptosis involves a combination of pyroptotic, apoptotic and necroptotic aspects of cell death [68].

## 3. Regulated Cell Death in Fungi

### 3.1. Why Should Fungi Undergo Regulated Cell Death?

It seems strange that programmed cell death should have evolved in single-celled organisms since loss of the whole organism would prevent the inheritance of pro-cell death genes to offspring unless the gains were indirect, benefitting closely related individuals [69]. Apoptosis-like cell death was detected in the slime mold, *D. discoideum* and parasitic *Trypanosoma* and *Leishmania* species and it was suggested that RCD of some *D. discoideum* cells to produce the stalk of the fruiting body enhances the dispersal of closely related spores while apoptosis-like RCD among parasitic protists is a way of avoiding a pro-inflammatory reaction that would threaten the survival (and therefore reproduction) of closely-related individuals in the host body (reviewed by [70]). It has been suggested that the RCD machinery was acquired by the ancestors of modern eukaryotes from the endosymbiotic proteobacterial and cyanobacterial ancestors of modern mitochondria and chloroplasts respectively and that modern RCD programs and complex apoptotic machinery arose from an arms race between the eukaryote and endosymbiont. The two participants had developed an “addictive” relationship and targeting the endosymbionts/mitochondria with pore-forming proteins became a means of self-destruction (apoptosis). The endosymbiont may have evolved the means to kill the host cell when conditions deteriorated (e.g., Bax/Bak), and the host evolved ways to inhibit this destruction (e.g., Bcl-2/Bcl-X_L_). There is also some evidence to suggest that most cell death proteins also have cell survival roles and did not evolve specifically for the purpose of mediating apoptosis. Büttner et al. [71] suggested that there are several good reasons why a single-celled organism should evolve RCD, including the ability to kill unrelated individuals and thus benefit kin, the ability to remove sexually incompatible individuals from the population and the ability of older, less fit cells to undergo cell death, sparing environmental nutrients, and releasing their own cell contents, to help power growth of fitter kin.

### 3.2. Early Examples of RCD in Fungi

#### 3.2.1. Unbalanced Growth

In the mid-20th century, it was shown in several fungal species, including *Ophiostoma multiannulatum, Neurospora crassa* and *Aspergillus nidulans*, (Table 1) that when some mutant strains were deprived of a particular nutrient, they underwent regulated cell death (RCD) due to an imbalance in metabolism, while others did not [72,73,74,75]. Deleting another gene, which affected a different branch of metabolism, appeared to restore balance and rescue the mutant strain from RCD.

#### 3.2.2. Heterokaryon Incompatibility

**Figure 8:**
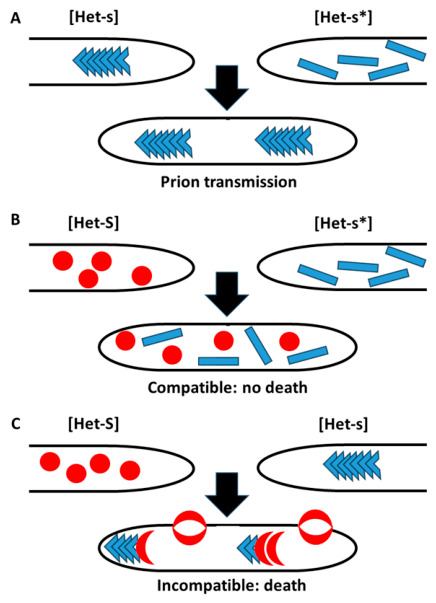
Heterokaryon incompatibility in *P. anserina*. Two mycelia fuse to form a heterokaryon but a cross between incompatible mycelia results in death of the heterokaryon and surrounding cells. Het-s*: strain produces soluble s protein (blue rods); Het-s—strain produces s prion (stacked blue chevrons); Het-S—produces S protein (red circles). Het-s prions proliferate in offspring of Het-s* x Het-s cross (**A**). Het-s* x Het-S—no prion involved so compatible (**B**). Cross of incompatible Het-s and Het-S—Het-s prions alter conformation of Het-S protein (red crescents), exposing transmembrane domain and leading to pore formation (two crescents forming a circle) and death of heterokaryon (**C**). Adapted from [78]. Creative Commons Attribution License.

In 1952, Professor George Rizet reported that crossing strains of *Podospora anserina* (one with an S allele, the other with an s allele) yielded S and S^s^ but no s offspring, that the S^s^ individuals were compatible with either parent and that the S^s^ strain sometimes reverted to the s genotype, which then grew to dominate the culture [76]. We now know this phenomenon as heterokaryon incompatibility (HI—a form of non-self-recognition), which is governed by the *het-S*/*het-s* locus [77,78]. When incompatible mycelia fuse, they form a heterokaryon and the resulting fusion cell and surrounding cells die via vacuolar lysis. The *het-S*/*het-s* gene product exists in three forms—HET-S, HET-s and HET-s*—and the HET-s form is a prion-forming protein while the HET-s* form is soluble. Crossing the het-s* strain with *het-s* produces offspring, harboring the HET-s prion (stacks of blue squares, Figure 8A). The het-s* and *het-S* strains are compatible and produce healthy offspring (Figure 8B). However, when *het-s* is crossed with *het-S*, the prion alters the conformation of the HET-S protein, exposing a transmembrane domain. HET-S forms pores in the membrane, triggering cell death (Figure 8C). HI has also been demonstrated in *Cochliobolus heterostrophus* [79,80,81,82], *Cryphonectria parasitica* [83] and *Neurospora* spp. [84,85,86,87]. HI-induced cell death involves DNA damage, shrinking of the cytoplasm and the formation of apoptotic bodies, which are hallmarks of apoptosis [88]. Numerous vacuoles appear, which may be autophagosomes—typical markers of autophagy and autophagic cell death. Some morphological changes (e.g., chromatin condensation and nuclear fragmentation) have been observed during both apoptotic and autophagic cell death. Heterokaryon incompatibility has been shown to limit the spread of mycoviruses and selfish genes between different populations of fungi as well as inhibiting the plundering of precious resources [89]. Many fungi possess multiple families of NOD-like receptor proteins and related proteins that are known to have roles in immune responses and cell death, and which mediate amyloid forming domain-related RCD that resembles necroptosis in animals and the hypersensitive response in plants. Similarly, gasdermins mediate immune-related pyroptotic cell death by forming pores in the cell membrane following cleavage of gasdermins by activated caspases in the pyroptosome. In *N. crassa,* activation of gasdermins occurs when gasdermins from non-compatible strains fuse to produce a pore-forming dimer. Interestingly, most fungal gasdermin genes cluster with protease genes and are activated proteolytically.

#### 3.2.3. Ascospore Abortion

**Figure 9:**
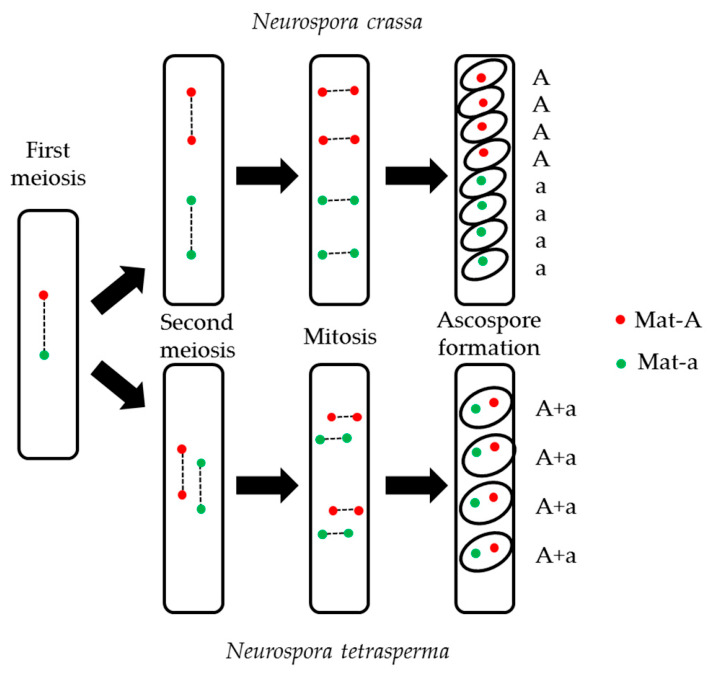
Spore development in *Neurospora* species. Following fusion of Mat-A and Mat-a gametes to form diploid zygotes, both *N. crassa* and *N. tetrasperma* undergo two rounds of meiosis and one of mitosis prior to ascospore formation. Mat-A and Mat-a nuclei segregate to opposite ends of the ascus during meiosis II in *N. crassa* but in *N. tetrasperma* the spindles overlap and nuclei of opposite mating types remain close together. Furthermore, *N. crassa* produces eight haploid ascospores while *N. tetrasperma* produces four diploid ascospores. *N. crassa* therefore produces ascospores with nuclei from only one parent while *N. tetrasperma* produces spores with nuclei from both parents. Adapted from [97] with permission from Oxford University Press.

Ascospore abortion has been documented in many different species, including *Podospora anserina* [90,91,92]. In *P. anserina* and *P. comata,* ascospore abortion is driven by the interaction of two genes: *het-S* and *het-s*, the latter of which encodes a protein product that can form a prion, triggering the death of *het-S* spore. *Neurospora* spp. undergo two rounds of meiosis, followed by a round of mitosis to form eight spores and, in a cross between spore-killer (*Sk*) and *Sk*-sensitive cells, half of the spores die [23,24,25,26,27,28,29,30,31,32,33,34,35,36,37,38,39,40,41,42,43,44,45,46,47,48,49,50,51,52,53,54,55,56,57,58,59,60,61,62,63,64,65,66,67,68,69,70,71,72,73,74,75,76,77,78,79,80,81,82,83,84,85,86,87,88,89,90,91,92,93,94,95,96,97,98,99]. Once the spore nuclei divide, sensitive cells lose their typical vacuolated appearance, the cytoplasm becomes disordered and the nuclei begin to break down [95]. Possession of *rsk* (resistance to spore killer) genes determines whether a spore will survive but *rsk* genes are specific to one or more *Sk* genes [99]. These two early observations of fungal regulated cell death (RCD) are examples of meiotic drive elements, which are overrepresented among offspring of a mating, at the expense of other alleles (Table 1). *N. crassa* possesses two different killer genes, *Sk-2^K^* and *Sk-3^K^*, and both the killer genes and resistance genes are only common in a few sites in Indonesia and Papua New Guinea. *N. tetrasperma* normally forms four ascospores. When the two *N. crassa* killer genes were transferred to *N. tetrasperma*, the *Sk-2^K^* x *Sk-3^K^* cross produced four heterokaryotic spores. However, when the eight-spore gene, *E* was also transferred to *N. tetrasperma*, the *Sk-2^K^* x *Sk-3^K^* cross yielded four live spores and four dead ones. During the second meiotic division, the nuclei from a- and α-mating type parents migrate to opposite ends of the ascus in *N. crassa* and each of the eight nuclei contains either a Mat-A or a Mat-a mating type nucleus (Figure 9). On the other hand, in *N. tetrasperma,* Mat-A- and Mat-a nuclei divide, parallel to one-another and only four spores are formed, so each spore contains one Mat-A and one Mat-a nucleus. This strategy of producing four spores with one nucleus from each parent is termed pseudohomothallism and occurs in *Gelasinospora tetraspora* and *Agaricus bisporus*. Sometimes, one of the expected Mat-A/Mat-a (A+a) spores is replaced by one Mat-A (A+A) and one Mat-a (a+a) spore, after the nuclei segregate in unexpected ways.

**Figure 10:**
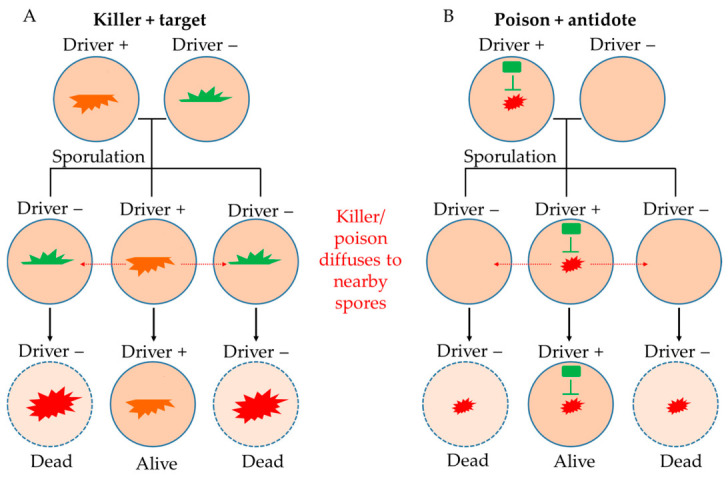
Spore killing mechanisms. In the killer + target mechanisms (**A**) one gene encodes the killer protein (orange semi explosion) and another the target protein (green semi explosion). If one or more spores inherit a killer gene (the driver), all spores are exposed to it but only spores that inherit the target gene die (whole red explosion). In the poison + antidote mechanism (**B**) the poison (red explosion symbol) and antidote (green square) proteins are encoded in the same gene (or overlapping genes). If one or more spores inherit this gene/these genes (the driver), all spores are exposed to the poison but only spores that inherited the driver benefit from the antidote and spores without the driver die. Adapted from [92] with permission from the American Society for Microbiology.

Two types of spore killing mechanisms have been described [92]—the “killer + target” and the “poison + antidote” systems. The killer + target mechanism involves a killer gene and a target gene, and only spores with the target gene are killed (Figure 10A). The poison + antidote mechanism involves a gene that encodes both a poison and its antidote (Figure 10B) and spores that possess the gene are protected from the poison by the antidote. The Sk system in *Neurospora* is an example of the killer + target mechanism while the Spok genes of *P. anserina* and *P. comata* and the *wtf* genes of *S. pombe* are examples of the poison + antidote mechanism. The Spok protein product acts as both poison and antidote while the wtf locus encodes overlapping genes, encoding a Wtf^poison^ and a Wtf^antidote^ protein [100,101]. There are many different wtf genes and the antidote, encoded in a particular wtf locus is specific to the poison, encoded at the same locus.

Other species that have been shown to undergo ascospore abortion include *Schizosaccharomyces pombe* [92,100,101], *Venturia inaequalis* [102], *Fusarium verticillioides* [103,104,105,106], *Bipolaris maydis* [107,108] and *Coniochaeta tetraspora* [109].

#### 3.2.4. Fruiting Body Development

Lu [110] reported that, during the development of fruiting bodies in *Coprinopsis cinereus*, the nascent gills begin as ridges, then cells between ridges break down, leaving pieces of cells and membranes, hydrolytic enzymes and multivesicular bodies. However, others have suggested that these markers of RCD were artifacts, resulting from the preparation of tissue for electron microscopy [111]. However, during fruiting body development, a kind of autolytic cell death removes gills that might be an obstacle to spore release, splits gills to allow opening of the cap or facilitates the release of copious amounts of extracellular matrix, which drives differentiation [112,113,114]. RCD is important in fruiting body development in many other species, including *Agaricus bisporus, Psilocybe* spp., *Panaeolus* spp., *Stropharia rugosoannulata*, *Coprinellus domesticus*, *Candolleomyces candolleanus, Candolleomyces candolleanus, Tremella mesenterica, Otidea onotica* and *Peziza ostracoderma* [112,113,114].

### 3.3. RCD in Saccharomyces cerevisiae

#### 3.3.1. Yeast Killer Toxin

**Figure 11:**
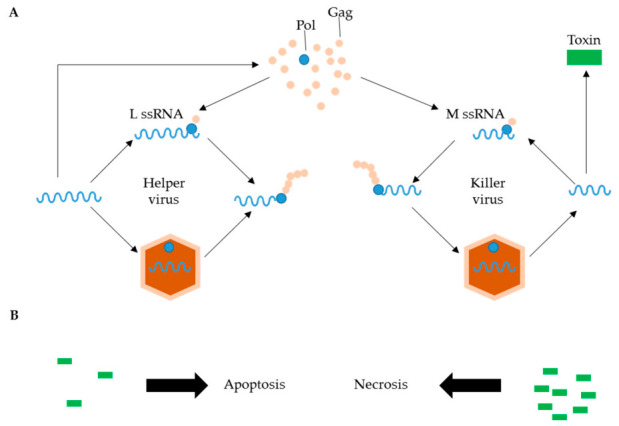
Yeast killer toxin. (**A**). Killer strains produce medium (M) RNA that encodes both the toxin and antidote (green square). A helper virus produces long (L) RNA, encoding both the Gag protein that makes up the viral capsule and Pol (polymerase). The killer and helper viruses both use Pol and Gag to build capsules and to replicate and maintain RNA. (**B**). Low concentrations of toxin induce cell death via apoptosis while high concentrations via necrosis. Adapted from [119] with permission from Springer Nature.

At low pH, some strains of *S. cerevisiae* secrete a substance that kills susceptible strains of yeast or closely related species [115,116,117,118,119] but are immune to the effects of this substance themselves. Following filtration, the medium in which killer yeast were grown retains the capacity to kill susceptible strains [115]. Treatment of a non-susceptible, killer strain with cyclohexamide converts it into a susceptible, non-killer strain [116], suggesting that a protein product of a yeast gene is required for production of the killer toxin. The killer yeast strain produces a 32 kDa protein, M-P1, which includes the killer toxin [117], and a non-killer, non-susceptible strain was shown to produce a similarly sized protein, implying that this strain has a defect in the dsRNA, encoding the protein, or in a host gene that is necessary for expression of the killer toxin. Killer yeast contain long (L) and medium (M) dsRNA, encoding the viral capsule and encapsulation machinery and the toxin and resistance proteins respectively [118]. It is now known [119] that the M dsRNA is produced by a killer virus (ScV-M1, ScV-M2 and ScV-M28) and encodes both a toxin and resistance factor (Figure 11) while the L-A dsRNA is produced by a helper virus ScV-L-A and encodes the Gag (capsule protein) and Gag-Pol (polymerase necessary for viral maintenance/encapsulation, etc.). In the case of K28 toxin, a high dose leads to cell death via necrosis (i.e., membrane disruption) while a low dose triggers cell death with hallmarks of apoptosis (membrane blebbing, PS flipping, etc.). The ca. 20 kDa K28 toxin enters the nucleus and disrupts the cell cycle by interfering with certain proteins.

#### 3.3.2. Sugar

Stationary phase *S. cerevisiae* cells lose viability when cultured in media containing hexose sugars (such as glucose or fructose) but lacking other nutrients, whereas there is no loss of viability in water or in media containing all nutrients except sugar [120,121,122,123]. “Sugar-induced cell death” requires phosphorylation of glucose (or fructose) but non-fermentable carbon sources, such as ethanol or acetate, generate phosphorylated sugars via glycolysis and these can then trigger cell death.

When exponentially growing cells were cultured in the presence of glucose (but not other nutrients), S-phase cells underwent programmed necrosis [124,125], as demonstrated by the permeability of the cell membrane to PI and FITC-labeled dextran (FD). This loss of membrane integrity was not secondary to apoptosis, since cycloheximide did not counteract this effect. Furthermore, annexin V and DAPI staining showed that exponential cells undergoing SICD did not resemble apoptotic (hydrogen peroxide-treated) cells with regards to PS flipping and chromatin fragmentation respectively.

Parbhudayal and Cheng [126] reviewed sugar-induced cell death in yeast. They compared SICD in log phase and stationary phase yeast with mammalian apoptosis and necrosis. All these forms of RCD involve increased mitochondrial outer membrane permeability and accumulation of ROS. Apoptosis and SICD in stationary phase yeast involve DNA damage, nuclear fragmentation and flipping of PS to the outer leaflet of the plasma membrane. However, primary necrosis and SICD in log phase yeast, but not apoptosis and SICD in stationary phase yeast, involve swelling of the nucleus. It was shown that necrosis and SICD in log phase yeast do not require biosynthesis of new proteins, whereas apoptosis does. Unfortunately, the requirement for protein biosynthesis had not been tested in stationary phase yeast SICD. It seems that SICD in stationary phase yeast cells resembles mammalian apoptosis while SICD in log phase yeast resembles primary necrosis.

SICD involves ROS accumulation and blocking ROS production, or treating cells with antioxidants, counteracts SICD but not completely, suggesting that there may be a ROS-independent pathway operating as well [123,125,127,128]. SICD also involves a reduction in cell membrane potential, which can be counteracted by addition of potassium chloride [129]. It is possible that glucose phosphorylation induces TORC activity, which inhibits autophagy and leads to cell death [126].

#### 3.3.3. *dc48^S565G^* Mutation

**Figure 12:**
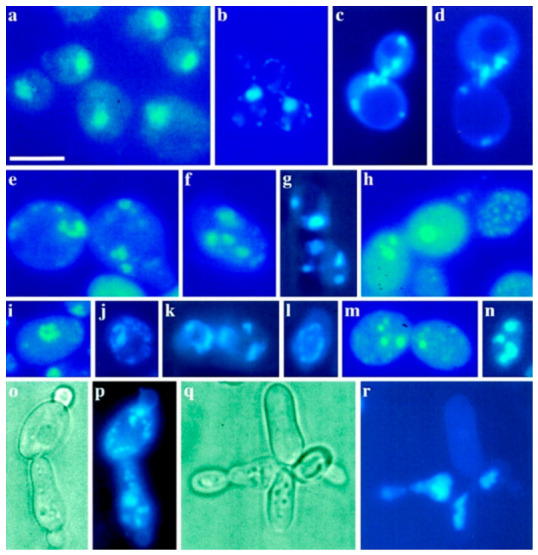
Chromatin fragmentation. DAPI-stained (**a**–**n**,**p**,**r**) and phase-contrast (**o**,**q**) images. All panels show strain KFY437, possessing a mutated/*cdc48^S565G^* gene, except negative control, which shows strain KFY417, with wild type *CDC48* (**a**) and positive control, which shows strain rE24-15, with a temperature-sensitive *cdc48-3^ts^* gene (**b**). Cells, grown on YEPD medium and harvested during log phase (**i**–**k**) or in stationary phase after 2 days (**a**,**c**–**h**,**l**–**n**), or after 5 days (**o**,**p**,**q**,**r**). Log phase rE24-15 cells were incubated at 37 °C for 3 h to arrest cell cycle (**b**). Bar—10 μ. Reproduced from [130] with permission from Rockefeller University Press.

**Figure 13:**
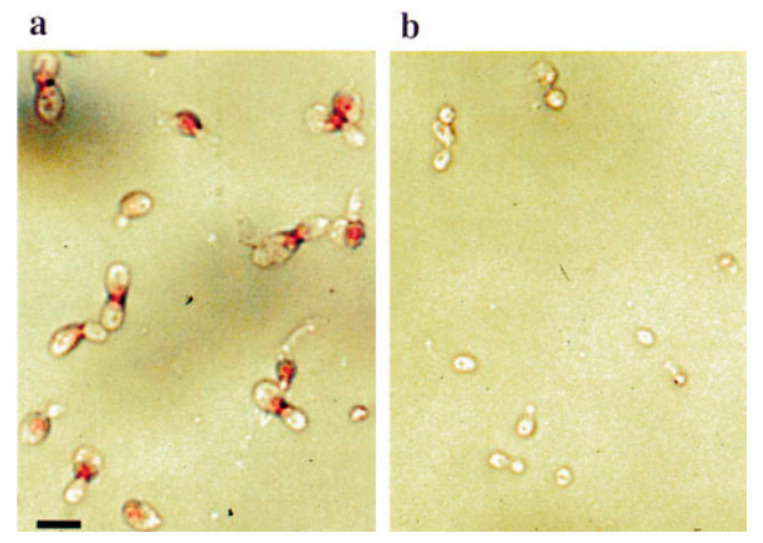
DNA strand breaks. Strain KFY437, with *cdc48-3^ts^* mutant gene (**a**) and negative control strain KFY417, with wild type CDC48 gene (**b**) were grown on YEPD for 36 h (to end of log phase); then, TUNEL staining was used to identify DNA strand breaks. Bar—10 μ. Reproduced from [130] with permission from Rockefeller University Press.

**Figure 14:**
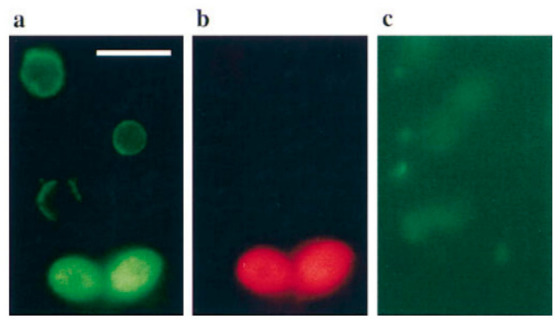
Flipping of phosphatidylserine. Strain KFY437, with *cdc48-3^ts^* mutant gene (**a**,**b**) and negative control strain KFY417, with wild type CDC48 gene (**c**) were grown on YEPD for 12 h then stained with FITC-labeled annexin V to identify flipping of phosphatidylserine from the inner to the outer layer of the plasma membrane (**a**,**c**) and with propidium iodide to identify cells with plasma membrane damage (**b**). Bar—10 μ. Reproduced from [130] with permission from Rockefeller University Press.

In 1997, Frank Madeo and coworkers [130] reported on a *Saccharomyces cerevisiae* strain with a mutation in the cell cycle gene *CDC48*—serine 565 was mutated to a glycine residue in the temperature-sensitive mutant. The mutation led to cell cycle arrest and the group found that arrested cells bore hallmarks of apoptosis (Figure 12, Figure 13 and Figure 14). DAPI staining of mutant cells from both 2-day and 5-day-old cultures revealed chromatin fragmentation (Figure 12), which was much less prevalent in wild type cells from stationary culture (Figure 12a) and highly pronounced in the *cdc48^S565G^* mutant, incubated at 37 °C for 3 h (Figure 12b), and in *cdc48^S565G^* mutant cells from stationary cultures (Figure 12c,n,p,r). They used TUNEL staining to reveal extensive DNA strand breaks in the mutant strain (Figure 13a) compared to the wild type control (Figure 13b). They also used annexin V staining to show that phosphatidylserine had flipped to the outer layer of the cell membrane in the mutant (Figure 14a) but not in the wild type (Figure 14c) and propidium iodide (PI) staining to show that some cells of the mutant strain had suffered membrane disruption (Figure 14b). Furthermore, electron microscopy revealed extensive chromatin condensation and fragmentation of nuclei in the *cdc48^S565G^* mutant strain, compared to the wild type.

#### 3.3.4. *Bax* Expression

During a yeast two-hybrid screen to identify interactions among human Bax and other Bcl-2 family members, it was found that a fusion protein containing Bax killed yeast and that another fusion protein containing Bcl-2 counteracted Bax-dependent killing [131]. Greenhalf and co-workers [132] showed that Bax arrests cell growth but that this only results in cell death when functional mitochondria are present. These findings are important because they indicate that the pro-cell death role of Bax and the anti-cell death roles of the Bcl proteins might be conserved in yeast and that apoptosis in yeast might resemble metazoan intrinsic apoptosis. Another group [18] showed that expressing Bax in yeast under the control of the *GAL10* promoter results in reduced levels of cytochrome c oxidase in mitochondria and release of cytochrome c but this effect is counteracted if Bcl-x_L_ (the longer splice variant of Bcl-x and a member of the Bcl-2 family) is also expressed. Overexpressing mammalian Bax in yeast produces changes in cell morphology that are typical of apoptosis—flipping of PS, chromatin condensation, membrane blebbing and DNA strand breaks [133]. Vacuolation of the cell during Bax-induced yeast cell death resembles that observed when Bax is overexpressed in human cells while caspase activity is inhibited [134]. This suggests that Bax-induced yeast cell death may resemble RCD in a common ancestor of yeast and humans, before the evolution of caspases. Xu and co-workers proposed using *S. cerevisiae* or *S. pombe* as models of Bax-induced PCD and published detailed protocols for expressing Bax in the fungal species and for detecting cell death [134]. Interestingly, deleting the N-terminal acetyltransferase component gene *NAT1* shifts acetic-acid-induced, human bax-dependent cell death in yeast from apoptosis to necrosis and it has been suggested that this is due to the role in acetylation of pro- and anti-apoptotic proteins and the subsequent effect on their activities [135].

#### 3.3.5. Oxygen Stress

Exposing yeast to oxygen stress via treatment with low concentrations of hydrogen peroxide (H_2_O_2_) or by deleting the glutathione (antioxidant) gene, led to enhanced apoptosis, as confirmed by TUNEL and DAPI staining and by electron microscopy [136]. Higher concentrations of H_2_O_2_ did not induce apoptosis but triggered loss of membrane integrity, as confirmed by PI staining and treating cells with cycloheximide abolished the pro-apoptotic effect of low-dose H_2_O_2_, indicating that new protein synthesis was required for this process. Furthermore, apoptosis in the temperature-sensitive *cdc48* mutant (above) and apoptosis in cells, expressing bax, was accompanied by the accumulation of ROS, and the addition of ROS scavengers (or growth under anaerobic conditions) reduced the incidence of apoptosis. Many cell-death-inducing agents trigger increased ROS accumulation, implying that ROS and mitochondrial dysfunction may be key players in RCD [137,138,139,140].

#### 3.3.6. Acetic Acid

It was reported as early as 1989 [141] that acetic acid induces two kinds of cell death: a low-enthalpy death at high acetic acid concentration and low temperature and a high-enthalpy death at high temperature and low acetic acid concentration. Prudêncio and coworkers [142] monitored Fun1p processing and PI staining to show that high concentrations of acetic acid reduce vacuolar processing and plasma membrane integrity. Ludovico and co-workers [143] showed that low-dose acetic acid induced apoptosis in yeast while a higher dose led to necrosis, as confirmed using annexin V, TUNEL and PI staining and electron microscopy. Low-dose, apoptotic cell death (measured by counting CFUs) was blocked by cycloheximide but high-dose, necrotic cell death was not. For technical reasons, it was not possible to study the effect of cycloheximide on PS flipping or DNA strand break formation. It was further shown [144] that acetic-acid-induced cell death in yeast involved ROS accumulation, activation of the proteasome then loss of cytochrome c from the mitochondria. Two pathways of acetic-acid-induced cell death were described—one was inhibited by N-acetyl cysteine (a scavenger of ROS) while the other was not (reviewed by [145]). Expressing human BRCA2, a protein involved in DNA repair via homologous recombination, enhanced cell death in response to acetic acid stress but not in the absence of acetic acid [146]. Heterologous expression of BRCA2 also led to upregulation of glycolysis and downregulation of TCA cycle and oxidative phosphorylation. In addition, BRCA2 inhibits the cell cycle during acetic-acid-induced cell death.

#### 3.3.7. Osmotin

The tobacco defense compound osmotin was shown to induce RCD in *S. cerevisiae* in a ROS- and Ras2-dependent manner [147]. The authors demonstrated that osmotin-induced ROS accumulation, DNA strand breaks and nuclear fragmentation as well as loss of viability, shrinking of the cytosol, vacuolation, membrane blebbing and formation of apoptotic bodies. Many of these are typical markers of apoptosis.

#### 3.3.8. Aging

Deletion of antioxidant genes or increased oxygenation leads to reduced replicative lifespan (RLS) while the antioxidant glutathione increases RLS [148]. Laun and coworkers [148] showed that replicatively old cells accumulate mitochondrial ROS and exhibit signs of apoptosis, such as DNA strand breaks and PS flipping. Chronologically, old cells accumulate ROS, lose viability and exhibit DNA strand breaks, PS flipping and chromatin condensation [149,150]. Furthermore, adding dried secretions from aged cultures to 7-day-old stationary cultures (but not log-phase cultures) led to an eight-fold increase in cell survival, while secretions from log-phase cultures had a much lower effect. This suggests that aging cells release substances during cell death that promote the survival of more viable cells. In aging yeast colonies on solid agar [151] the production of ammonia by colony cells triggers RCD of central cells, releasing nutrients for use by outer cells. Central cells display elevated levels of ROS accumulation, DNA strand breaks, DNA fragmentation and cell shrinkage than outer cells, taken from the same colonies. In colonies of a *sok2* knockout mutant, unable to produce ammonia, outer cells were subject to significant levels of RCD too, implying that the ammonia-induced death of central cells released nutrients that were used by outer cells. Inner cells died despite being closer to available nutrients in the agar, just as outer cells downregulated oxidative phosphorylation, despite having better access to air, suggesting that the greater age of inner cells might contribute to RCD that benefits replicative outer cells [152].

#### 3.3.9. Pheromone

A low dose of alpha-factor pheromone (produced by yeast cells of mating type α) induced shmoo formation and mating in cells of mating type a [20]. A dose, ten times higher, induced RCD in unmated a-type cells, involving an increase in ROS, DNA strand breaks, DNA fragmentation and membrane disruption. Blocking protein biosynthesis or opening of the permeability transition pore (PTP) or knocking out the MAP kinase component gene, *STE20*, counteracted this effect while deleting genes, encoding components of the calcineurin/calmodulin system, rendered yeast even more sensitive to pheromone-induced cell death. Deleting *LAM2*, one of four genes with roles in the transport of sterols from the plasma membrane to the endoplasmic reticulum, partially counteracts pheromone-induced cell death in *S. cerevisiae* strain W303 [153]. The loss of *LAM2* or *LAM1* reduces sterol internalization, leading to sterol accumulation in the plasma membrane and thus cell death.

#### 3.3.10. Other Stimuli That Trigger Yeast RCD

Other factors that induce RCD in yeast (Table 1) include sodium chloride [154], defects in mRNA decapping [155], aspirin [156,157,158], hypochlorous acid [159,160,161], defects in DNA replication [162,163,164], hyperosmotic stress [165,166,167], reduced sister chromatid cohesion via *PDS5* mutation [168,169], defects in N-glycosylation due to *OST2* or *WBP1* deletion or in temperature-sensitive mutants of either gene at 37 °C [170], copper or manganese [171,172,173], formic acid [174,175], palmitate-induced ER stress [176,177], accumulation of Ras2 in mitochondria due to deletion of *WHI2*, HXK2 or *SNF1* [178,179,180,181], low availability of potassium [129,182,183], a lack of H2B K123 ubiquitination [184,185,186], exposure to lead [187,188], the epidermal growth factor receptor (EGFR) antagonist gefitinib [189], cisplatin [190], heat shock [191,192,193], anacardic acid [194,195,196], cold plasma [197,198], nickel oxide nanoparticles [199,200,201,202], citral or geraniol [203,204,205], expression of human caspase-1 in yeast [206,207], defects in mitophagy or autophagy due to deletion of *PIL1* [208], synthetic antimicrobial peptides [209], oxidative stress induced by polyethylene terephthalate Nano plastic [210,211], silver nanoparticles [212], cohesion dysfunction [170], Deletion of AP-3 components or downstream kinase [213], and enhanced mitochondrial DNA damage due to *HAP4* deletion [139].

### 3.4. RCD in Schizosaccharomyces pombe

Expressing Bax or Bak in *S. pombe* led to RCD, which was characterized by vacuolarization of the cytoplasm, DNA condensation and disintegration of the nuclear envelope and RCD was mediated via cell cycle arrest and was abrogated by Bcl-2 or Bcl-X_L_ [214,215,216]. Deleting either of two genes, involved in the last step of diacylglycerol biosynthesis causes *S. pombe* cells to undergo RCD upon the onset of stationary phase [217,218,219,220]. TUNEL staining showed increased DNA strand breaks, DAPI staining of the nucleus confirmed increased DNA fragmentation, annexin V staining revealed flipping of PS in the plasma membrane and PI staining showed an increased incidence of cells with membrane damage in the mutant strains, compared with the wild type. Mutations in DNA synthesis initiation genes leads to the accumulation of ROS and cell death and delaying replication fork progress via treatment with hydroxyurea increases ROS accumulation and cell death [221,222]. Inositol starvation induces cell death in fission yeast that is dependent on the chaperone calnexin [223,224]. Chronological aging also causes ROS accumulation and cell death in *S. pombe* and deleting *SCH9* or *PCA1* partially counteracts this effect while dysfunctional antioxidant defenses exacerbate the effect [220,225].

### 3.5. RCD in Human Fungal Pathogens

#### 3.5.1. *Candida albicans*

Low doses of acetic acid, hydrogen peroxide and amphotericin B elicit RCD in *C. albicans* with hallmarks of apoptosis, including PS flipping, DNA strand breaks, chromatin condensation, nuclear fragmentation and ROS accumulation [226]. Higher doses elicited necrotic cell death. Cell death was later shown to be mediated by Ras-cAMP-PKA signaling and blocking this signaling pathway delayed cell death while increasing Ras-cAMP-PKA activity accelerated RCD [227,228,229,230]. Another antifungal drug, caspofungin induces cell death in *C. albicans* [231,232,233]. At low doses, about 25% of cells showed hallmarks of apoptosis (DNA strand breaks, ROS accumulation, loss of mitochondrial membrane potential, chromatin condensation and blebbing of the nucleus) while a smaller percentage stained with PI, implying necrosis. The fraction of necrotic cells increased at higher concentrations of the drug. Amphotericin B was shown to induce RCD in *C. albicans* even when cells were embedded in the extracellular matrix of biofilms and *Pseudomonas aeruginosa* increased the susceptibility of *C. albicans* biofilms to this drug [234,235] and there was some evidence that histone acetylation might promote AmB-induced RCD. *C. albicans* secretes the quorum sensing messenger farnesol, which inhibits the switch to hyphal morphology at physiological concentrations but induced RCD in *C. albicans* at high concentrations (100 µM) [236,237]. It was shown that cells upregulated heat shock and antioxidant protein expression while downregulating metabolic enzyme expression. Treated cells died with hallmarks of apoptosis (DNA strand breaks, ROS accumulation and mitochondrial fragmentation, etc.). The plant metabolite, aureobasidin A, promotes *C. albicans* RCD by inhibiting inositol phosphorylceramide synthase, which leads to altered membrane fluidity, reduced membrane potential and increased ROS accumulation [238].

#### 3.5.2. *Aspergillus* Species

*Aspergillus fumigatus* undergoes rapid, widespread cell death on entry to stationary phase [239]. *Aspergillus nidulans* may undergo cell death, accompanied by caspase-like activity and loss of poly(ADP-ribose) during sporulation [240,241]. Farnesol induces RCD in other organisms, including *A. nidulans* [242]. Treating *A. nidulans* with farnesol, blocked hyphal formation and growth and induced condensation of the nucleus. It also induced DNA strand break formation, flipping of PS and accumulation of ROS, implying an apoptosis-like RCD. Clove and rosemary oil both induced RCD with hallmarks of apoptosis in the plant, animal and human pathogen, *A. flavus* [243,244]. Inhibition of the unfolded protein response enhanced antifungal drug susceptibility and drug-induced cell death in *A. fumigatus* [245]. The plant essential oil, perillaldehyde induced RCD in *A. flavus* by altering metabolism, leading to a scarcity of reducing equivalents and therefore increased accumulation of ROS [246]. Similarly, carvacrol, a constituent of plant essential oils, induced *A. niger* RCD via increased hydrogen peroxide accumulation, downregulated expression of NADPH oxidase, oxidative stress and peroxidation of lipids [247]. Furthermore, hexanal triggered RCD in *A. flavus* via loss of mitochondrial membrane potential, oxidative stress and DNA damage [248].

#### 3.5.3. *Cryptococcus neoformans* and *Histoplasma capsulatum*

Dadachova et al. [249] set out to test the possibility of treating fungal infections with radiation but fungal pathogens may be thousands of times more resistant to gamma rays than human cells. They attached radioactive isotopes to antibodies that targeted *C. neoformans* or *H. capsulatum* and showed that the former is 1000 times and the latter 100 times more sensitive to radioisotopes emitting alpha or beta radiation than gamma rays. Low dose alpha or beta radioimmunotherapy elicited apoptosis-like RCD in most cells of both species while high dose gamma rays produced similar changes in only 30% of cells.

### 3.6. RCD in Plant Fungal Pathogens

*Colletotrichum trifolii* expressing constitutively active Ras underwent RCD via ROS accumulation, PS flipping, DNA fragmentation, etc., when grown on minimal medium [250] but proline addition prevented RCD, possibly via ROS scavenging. Sodium chloride, heat shock, UV radiation and hydrogen peroxide also triggered RCD in *C. trifolii*. Barhoom and Sharon [251] found that expression of Bax in *Colletotrichum gloeosporioides* was lethal so they co-expressed Bax with inducible Bcl-2. Bcl-2 suppressed Bax lethality, but when Bcl-2 expression was switched off, fungal cells underwent RCD. Magnolol, a polyphenol extracted form magnolia, kills *C. gloeosporioides* by permeabilizing the plasma membrane, causing leakage of cell contents and leading to accumulation of ROS [252]. Zygocin, the yeast killer toxin produced by *Zygosaccharomyces bailii* was shown to inhibit aerial mycelia growth of the plant pathogens, *Colletotrichum graminicola* and *Fusarium oxysporum* [119,253], as well as several human fungal pathogens. Further investigation, using *S. cerevisiae*, showed that zygocin induced apoptosis-like RCD.

## 4. Caspase Orthologs and Other Death Protein Orthologs

**Figure 15:**
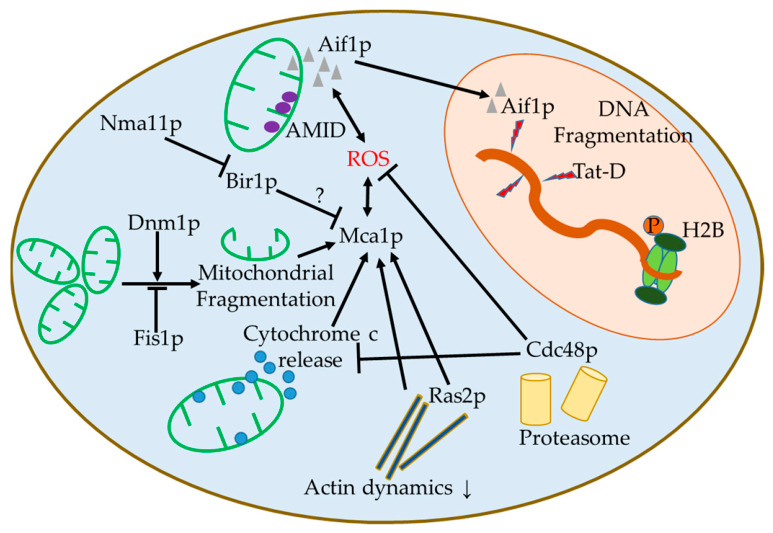
Yeast RCD mediators. Mca1p, ROS and cytochrome c release were shown to be involved in yeast RCD. The yeast inhibitor of apoptosis (IAP), Bir1p may inhibit Mca1p. The ortholog of Omi/HtrA2, Nma11p cleaves Bir1p. Cytochrome c release, mitochondrial fragmentation and reduced actin dynamics (via Ras2p) activate Mca1p. Mca1p activation, dysfunctional protein aggregate degradation and release of Aif1p promote ROS accumulation. Cdc48p, and the proteasome drive the degradation of protein aggregates, counteracting ROS accumulation and cytochrome c release. Aif1p promotes DNA strand breakage by the endonuclease, Tat-D. Phosphorylation of histone H2B has also been shown to promote chromatin condensation and DNA fragmentation. Arrow—activation/promotion; T-bar—inhibition/inactivation. Adapted from [71] with permission from Rockefeller University Press.

Orthologs of proteins with roles in metazoan RCD have been discovered in yeast (Figure 15), including orthologs of caspases (metacaspase Mca1p), cytochrome c, AIF/AMID (Aif1p and Ndi1p), IAP (Bir1p) and Omi/HtrA2 (Nma111p) [71,254,255]. It was shown that many yeast genes, encoding orthologs of mammalian apoptosis proteins could be substituted with gene orthologs from mammals, protists, plants and bacteria [256]. Examples include the *Arabidopsis thaliana* ortholog of Mca1p, the human ortholog of Nuc1p, the *Desulfatibacillum alkenivorans* ortholog of Nma111p and the *Dictyostellium discoideum* ortholog of Ndi1p, suggesting a broad conservation of cell death mechanisms and effectors across the tree of life.

It has been suggested that the eukaryotic RCD machinery, including caspases and caspase orthologs, may have been inherited from the bacterial symbiont that evolved into mitochondria [70] and that this development may have arisen from a mutual addiction relationship. Bacterial/mitochondrial genes have mostly been relocated to the host cell nucleus and are transcribed and translated by the host before migration to the mitochondria. Therefore, mitochondria cannot survive independently of the host cell, and eukaryotes rely on mitochondria for respiration. RCD effectors such as the pro-apoptotic Bcl-2 may have evolved from bacterial proteins that allowed the symbiont to kill the host cell when conditions became unfavorable. It has more recently been argued that the addiction hypothesis cannot be correct because bacteria do not possess orthologs of anti-apoptotic genes [257]. Johnson and Kranzusch [258] used the example of gasdermins, which are conserved among bacteria, fungi and mammals, to demonstrate the common role of regulated cell death in diverse organisms. Gasdermins are activated by cleavage and go on to form pores in the plasma membrane, leading to cell death. They have an important role in protection against viral infection, killing infected individuals to prevent the spread of a virus throughout the population.

### 4.1. Discovery of Metacaspases, Paracaspases and Orthocaspases

Multiple rounds of searches using the basic local alignment search tool (BLAST + 2.17.0) at the EMBL/EBI protein database [259], using a consensus sequence generated via multiple alignment of known caspases, identified distantly related proteins with significantly similar sequences, including conserved active site residues [19]. Further research was carried out using the sequence of this new caspase ortholog and a further consensus sequence, generated by another round of alignments. The newly discovered caspase orthologs included proteins from *Caenorhabditis elegans*, *Dictyostelium discoideum*, *Streptomyces coelicolor* and a *Rhizobium* species. Based on these findings, further rounds of iterative BLAST searches were carried out and identified three new families of caspase orthologs [17]. These families were paracaspases (in metazoa and *D. discoideum*), metacaspases (in plants, algae, fungi and protists) and distant caspase orthologs in bacteria, such as gingipain R in *Porphyromonas gingivalis*. More recently, various groups have identified proteases, termed “prokaryotic metacaspase-like proteases” or “orthocaspases” [26].

### 4.2. Structure of Caspase Orthologs

**Figure 16:**
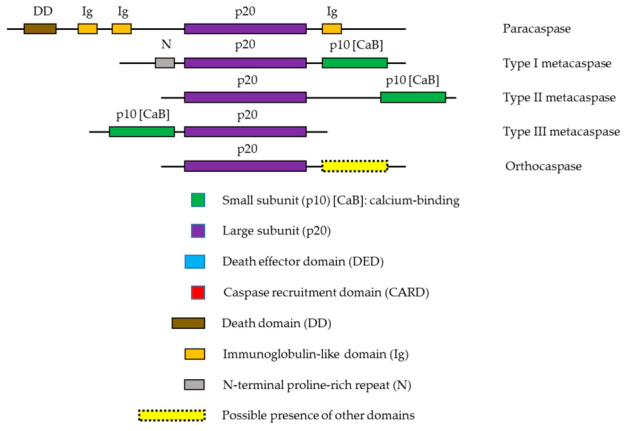
Structures of caspase orthologs—paracaspases and type I, II and III metacaspases differ in the presence of various domains. The small subunit is absent from paracaspases and may be absent from orthocaspases (metacaspase-like proteases). The positions of the small and large subunits are reversed in type III metacaspases. The structures of caspases-3, -8 and -9 are shown for comparison. The small subunits of metacaspases have calcium binding (CaB) domains while those of caspases do not. Adapted from [26] https://creativecommons.org/licenses/by-nc-nd/4.0/, Accessed on 27 May 2026.

The structures of type I, type II and type III metacaspases and paracaspases differ (Figure 16). Paracaspases (found in animals and slime molds) include immunoglobulin-like domains and death domains [260]. Type I metacaspases (found in fungi, protists and brown algae, etc.) have an N-terminal, proline-rich region and may or may not have a zinc finger motif [26,261,262]. Type II metacapases (found in plants and green algae) have a long linker region between the large and small subunits. Type III metacaspases (found in phytoplankton) have a large and small subunit but no protein interaction motifs [260]. It has been proposed that the presence of type I metacaspases in fungi, protists and brown algae, etc., but type I and type II metacaspases in plants may have originated via horizontal gene transfer of primitive metacaspases from the ancestors of modern mitochondria and chloroplasts respectively [263].

### 4.3. Mechanism of Metacaspase Activity

Like caspases, metacaspases have a large and a small subunit, with the catalytic Cys/His dyad in the hemoglobinase fold within the large subunit [264]. *Arabidopsis thaliana* metacaspases AtMC4 and AtMC9 were shown to cleave substrates with Arg or Lys at the P1 position rather than aspartate [261]. It was also shown that the glycine and arginine residues that form the basic S1 pocket of caspases (into which the acidic aspartate residue fits) are replaced by aspartate or glutamate residues in metacaspases, forming an acidic pocket into which the basic arginine fits [261]. Furthermore, the initial cleavage target lysine between the large and small subunits of AtMC4 is masked by N-terminal and C-terminal loops and calcium ions activate cleavage at this and other basic amino acid residues, separating the subunits and activating proteolytic activity [265]. Treatment of *Trypanosoma brucei* with calcium induced metacaspase MCA2 activation, which was blocked by the addition of the calcium chelation agent EGTA [266]. Similarly, activation of *A. thaliana* metacaspases AtMCP2a and AtMCP2b, as well as the yeast metacaspase Mca1p, requires relatively high concentrations of calcium, the concentration of which is increased during certain types of stress [267].

### 4.4. Metacaspase-Dependent RCD in Fungi

Many groups have demonstrated the activation of caspase-like proteases during RCD using fluorescent versions of pan-caspase inhibitors, e.g., FITC-VAD-FMK (fluoroisothiocyanate-labeled valyl-alanyl-aspartyl-[O-methyl]-fluoromethylketone) [162]. Chronological aging and killer toxins promote cell death via Mca1p [20,149,151,268], as does hyperosmotic stress, due to high glucose, sorbitol or sodium chloride concentration [165,269]. Valproic acid-, arsenic-, caffeine- and metal-ion-induced cell death are also Mca1p-dependent [171,270,271,272]. 

#### 4.4.1. Aging, Mca1p and Cell Death

Age-induced RCD in yeast is mediated by yeast metacaspase Mca1p [21,149]. It was shown that Mca1p was cleaved to remove a small subunit and thus activate the large subunit in a similar manner to mammalian caspases but mutation of catalytic cysteine 297 abolished cleavage. They further showed that a fluorescent initiator caspase substrate, Ac-IETD-AMC (acetyl-isoleucyl-glutamyl-threonyl-aspartyl-amino-4-methylcoumarin) was robustly cleaved by Mca1p while an executioner caspase substrate, Ac-DEVD-AMC (acetyl-aspartyl-glutamyl-valyl-aspartyl-amino-4-methylcoumarin) was not. In strain *mca1^C296A^*, in which the catalytic cysteine residue was mutated to alanine, caspase-like activity was not as high as in the wild type but was higher than in the empty vector control, which now seems questionable as the mutation of the cysteine should have abolished “caspase” activity (though not aspartyl proteases activity). The authors used TUNEL and DAPI (4′,6-diamidino-2-phenylindole) staining to show that hydrogen peroxide-induced cell death was accompanied by markers of apoptosis—breakage of DNA strands and condensation of chromatin respectively and that these were abrogated by treatment with cell-permeable pan-caspase inhibitor zVAD-fmk (carbobenzoxy-valyl-alanyl-aspartyl-[O-methyl]-fluoromethylketone) or by mutation of the catalytic cysteine to alanine. This indicated that Mca1p did play a role in linking apoptogenic stimuli with the downstream effects of RCD and with the increase in caspase activity, but cleavage of caspase substrates by Mca1p may be non-specific and the metacaspsase may act upstream of other activated aspartyl proteases.

Herker and coworkers [149] measured “caspase activation” during chronological aging using FITC-VAD-fmk (a fluorescent analog of a pan-caspase inhibitor) and flow cytometry. FITC-VAD-fmk fluorescence was higher at 3 days and 5 days than in 4-h-old cultures and deleting *MCA1* abolished most of this activity in 3-day-old cultures but had a much smaller effect on 5-day-old cultures. One possibility is that Mca1p plays a role in aspartyl protease activation during early RCD or when stress levels are below a particular threshold but is not as important once cells have committed to the cell death program. Increased cell death of aged cells is accompanied by the accumulation of activated metacaspase and Wang et al. [273] showed that Mca1p activity is reduced in younger cells via the production of reactive sulfane sulfur (RSS) by cystathionine-γ-lyase. RSS interacts with the catalytic cysteine of Mca1p, inhibiting autocleavage of the metacaspase and inhibiting cleavage of Bir1p, a known target of Mca1p. Deleting the gene for cystathionine-γ-lyase leads to a reduction in chronological lifespan.

#### 4.4.2. Salt and Osmotic Stress

Deleting *MCA1* alleviates symptoms of RCD, induced by sodium chloride in a mutant strain, deleted for tumor suppressor homolog *SRO7* [269]. “Caspase activity during apoptosis” was detected using FITC-VAD-fmk. RCD in response to sorbitol- or glucose-induced hyperosmotic stress was shown to be dependent on the presence of intact cytochrome c and Mca1p but independent of the apoptosis-inducing factor ortholog Aif1p [165]. Apoptosis-like cell death was confirmed using electron microscopy and TUNEL, DAPI and PI staining, as well as dihydroethidium (DE) or 2′,7′-dichloro-dihydrofluorescein diacetate (H_2_DCFDA) to measure reactive oxygen species (ROS). The authors also used a double staining technique involving FITC-VAD-fmk and PI to detect “caspase activity”. The fact that deletion of *MCA1* abrogates “yeast apoptosis” and that “yeast apoptosis” involves “caspase” activity did not necessarily show that Mca1p was responsible for the “caspase” activity detected. Stressed cells were examined using holotomography and it was found that cells under relatively mild osmotic stress became shorter and rounder and cell volume was reduced [274]. In addition, almost 2% of cells stained with PI, implying necrosis, and the total amount of proteins and lipids were severely reduced, as was the number of lipid droplets.

#### 4.4.3. Farnesol

Shirtliff et al. [236] found that during farnesol-induced RCD in *C. albicans*, *MCA1* expression was upregulated, that cells died with hallmarks of apoptosis (DNA strand breaks, ROS accumulation and mitochondrial fragmentation, etc.). They also found that farnesol induced caspase-like proteolytic activity in a dose-dependent manner. At the time, caspase activity was seen by many as a defining characteristic of apoptosis, and this may have influenced the enthusiasm with which some researchers sought to prove that RCD was accompanied by increased caspase activity. Unfortunately, the group did not report whether the FLICA kit they used detected pan-caspase or specific caspase-type activity. In *N. crassa*, mutations that negatively affects complex I activity reduce ROS accumulation and rescue the fungus from farnesol-induced cell death [275]. Overexpressing *NDI1* (encoding a key enzyme in the electron transport chain) in *A. nidulans* has no effect on ROS accumulation but leads to reduced cell death while *NDI1* overexpression in *S. cerevisiae* increases ROS levels and cell death. This suggests that responses to stressors and the roles of pro- and anti-apoptotic components of the cell machinery may differ in different organisms, cell types and conditions.

#### 4.4.4. Antifungal Drugs and Fungicides

Amphotericin B, caspofungin, fluconazole, itraconazole and micafungin all trigger RCD in fungi with hallmarks of apoptosis [276] and usually involve ROS accumulation, misfunctioning mitochondria and metacaspase activation. Hao and coworkers [231] used the multi-caspase substrate (aspartyl)_2_-Rhodamine 110 (D2R), cleavage of which releases fluorescent rhodamine, to show that caspofungin-induced RCD was accompanied by “caspase activity” in *C. albicans*. We now know that fungi do not possess bona fide caspases. Al-Dhaheri and Douglas [234] demonstrated caspase-like activity during amphotericin B-induced RCD in *C. albicans* biofilms, using both SR-FLICA (a poly-caspase substrate) and D_2_R. They also showed that caspase inhibitors, particularly those that blocked caspases-1, 2, 3 and 5, enhanced the survival of AmB-treated biofilm cells. The caspase inhibitor was Z-X-FMK, where X is a synthetic caspase substrate (e.g., YVAD) and is attached to a fluromethylketone (FMK) group, which covalently (and irreversibly) binds to the catalytic cysteine via its sulfur atoms, blocking enzymatic activity. There is no data on how the use of caspase inhibitors affected the level of aspartic protease activity during RCD. The agricultural fungicide, mancozeb, kills yeast via upregulated ROS production and increased mitochondrial membrane potential, eliciting alterations in yeast cells that are typical of apoptosis and are dependent on Mca1p [277].

### 4.5. Metacaspase-Independent RCD

#### 4.5.1. Sphingolipids

Dihydrosphingosine and phytosphingosine induced RCD in *A. nidulans* [240]. This involved ROS accumulation, but ROS scavenging did not abrogate RCD, suggesting that RCD was not dependent on ROS production. This sphingolipid-induced RCD resulted in increased PS flipping and DNA strand breakage and the latter required active protein synthesis, implying that cell death was programmed. The RCD process was not affected by *MCA1* deletion but required functional mitochondria.

#### 4.5.2. Altruistic Death of Yeast Cells in Colonies

The survival of outer cells in yeast colonies [151] at the expense of central cells (which undergo RCD) is independent of Mca1p and Aif1p. Ammonia is used as a messenger to regulate coordinated cell death among central cells. Colony cells, deleted for *MCA1* stain with D_2_R, indicated the presence of an active aspartyl protease. This could explain the many reports of aspartyl protease activity during yeast RCD, even after *MCA1* deletion or inhibition with z-VAD-fmk.

#### 4.5.3. Antimicrobial Peptides

A truncated (but active) version of the tree frog antimicrobial peptide, dermaseptin S3 was found to kill yeast, with increased ROS production, flipping of PS and DNA strand breakage [278]. Dermaseptin-induced cell death was dependent on Aif1p and on two proteins (Stm1p and Izh2p) that had been implicated in other forms of yeast cell death, but not on Mca1p.

#### 4.5.4. Acetic Acid

Mca1p was not essential for acetic-acid-induced RCD [144] since acetic acid induced two forms of RCD—an Mca1p-dependent and an Mca1p-independent pathway [145]. Deletion of *MCA1* did not abolish acetic-acid-induced RCD but reduced the rate of cell death. Furthermore, inhibition of Mca1p with z-VAD-fmk did not rescue WT or deletant cells from RCD, implying that neither form of cell death was dependent on the catalytic function of Mca1p.

#### 4.5.5. Metals

Nickel oxide nanoparticle-induced RCD in yeast was found to be dependent on Yca1p and Aif1p [199]. Copper-induced yeast RCD was also shown to be independent of Mca1p, involved production of copious ROS and was alleviated by the addition of antioxidants (unlike manganese-induced RCD) [171] Anacardic acid was shown to induce RCD in *S. cerevisiae* with hallmarks of apoptosis [194]. RCD was not suppressed by knocking out metacaspase gene *MCA1* or by the presence of caspase inhibitor Z-VAD-fmk, implying that anacardic acid-induced cell death in yeast is independent of Mca1p.

#### 4.5.6. Formic Acid

Low doses of formic acid induce RCD with hallmarks of apoptosis [174] including DNA strand breaks, chromatin fragmentation, nuclear condensation, flipping of PS, reduced potential in the mitochondrial membrane and accumulation of ROS. Deletion of *MCA1* resulted in an earlier and stronger burst of ROS production but *MCA1* deletion did not abrogate RCD.

#### 4.5.7. Caspofungin

The antifungal drug caspofungin blocks β-1,3-glucan synthesis, which is important for cell wall integrity, and elicits RCD in *S. cerevisiae* and *C. albicans* with ample ROS accumulation and DNA strand breakage [279]. Caspofungin-induced RCD was abrogated by *AIF1* deletion but not by *MCA1* deletion.

### 4.6. Non-Cell Death Roles of Yeast Metacaspase

#### 4.6.1. Clearance of Protein Aggregates

Mca1p has a non-RCD role, helping to clear protein aggregates [280]. The metacaspase was shown to localize to protein aggregates and interact with heat shock proteins in a way that was dependent on its polyglutamine-rich domain. Deletion of *MCA1* or altering the gene to express catalytically inactive Mca1p resulted in an increased buildup of aggregated proteins. It has been proposed that calmodulin signaling governs the switch from a death-inducing protease to a pro-survival mediator of proteostasis [281].

#### 4.6.2. Cell Cycle Control

Mca1p also appeared to play a role in regulation of the cell cycle. Deletion of *MCA1*, or mutation to abolish Mca1p catalytic activity, extended the G1/S transition of the cell cycle [282]. Furthermore, cells with reduced Mca1p catalytic activity did not arrest when treated with nocodazole (a microtubule inhibitor). This implied that Mca1p was also involved in the G2/M checkpoint. This phenomenon was not dependent on ROS production. Interestingly, metacaspases have also been implicated in growth and cell cycle regulation of the protist human parasites, *Trypanosoma brucei* and *Leishmania* major [283]. This makes metacaspases attractive targets for antifungal and anti-parasitic drugs.

## 5. The Debate

Fungi undergo RCD in response to various stimuli, sometimes with characteristics of metazoan apoptosis [71,284]. The discovery of caspase orthologs as well as orthologs of other metazoan cell death proteins in fungi suggested that fungi might possess similar cell death pathways [17]. Frank Madeo has been perhaps the greatest proponent of the theory that metacaspases are caspases, and named the yeast metacaspase gene “Yeast Caspase 1” (*YCA1*) after demonstrating its involvement in yeast RCD [21]. Some forms of yeast RCD are dependent on Mca1p and some are not [285,286], Mca1p has non-cell death roles [280,282], and some parasitic protists have metacaspases that regulate growth, the cell cycle and differentiation [283]. In line with caspases, and other caspase orthologs, metacaspases are clan CD, family C14 proteases (https://www.ebi.ac.uk/merops; Accessed 17 March 2026) with a characteristic active site (the hemoglobinase fold) and (with some exceptions) a catalytic cysteine/histidine dyad [261]. Metacaspases cleave peptides after arginine or lysine, whereas caspases cleave after aspartate [1]. Furthermore, metacaspases require calcium for activation [265].

### 5.1. Are Metacaspases Caspases?

Vercammen et al. [263] wrote an editorial entitled “Are metacaspases caspases?” Referring to the finding of Madeo’s team [21] that Mca1p mediates cell death, they remarked that upregulated stress following overexpression of a stress-inducing protease was no surprise. They further queried the Madeo et al. [21] report of RCD-related caspase activity as it was based on the use of unreliable synthetic caspase substrates, and Mca1p from cells undergoing hydrogen-peroxide-induced cell death had been shown to cleave after Arg or Lys, not Asp [267]. Vercammen et al. [263] went on to question the involvement of some metacaspases in RCD, particularly those with roles in development and growth.

### 5.2. Metacaspases Are Caspases, Doubt No More

In their editorial, (title above), Carmona-Gutierrez et al. [287] (Madeo’s team) acknowledged that caspases and metacaspases cleave at different residues but theorized that they would cleave similar substrates as part of a conserved cell death process. They went on to comment on the discovery that the Norway spruce (*Picea abies*) metacaspase, MCII-Pa cleaved Tudor staphylococcal nuclease (TSN) during RCD, induced by stress or related to development [288], and that human TSN was cleaved by caspase-3 during apoptosis. Human TSN is involved in splicing and transcription and cleavage of TSN led to reduced expression of some pro-survival mRNAs and this resulted in cell death. Madeo and his team [287] argued that this was evidence that caspase substrates, and therefore the cell death regime, was conserved in yeast.

### 5.3. Metacaspases Are Not Caspases—Always Doubt

In response, Enoksson and Salvesen [289] wrote an editorial with the title shown above. They begin by agreeing that the naming of caspase orthologs as metacaspases and paracaspases was confusing and then stated that it was unscientific to suggest there was no doubt about the relationship between caspases and metacaspases since nothing can be proved in science, only disproved. They went on to say that apoptosis arose only in multicellular organisms, and that MCII-Pa cleaves PaTSN in four different places instead of one specific place, thus resembling protein degradation rather than cell-death-related cleavage. Enoksson and Salvesen [289] argued that the definition of caspases (cysteine dependent aspartyl proteases) specifically stipulates cleavage after aspartate and therefore excludes metacaspases.

## 6. Assay Methods for Detecting Fungal RCD

Generally, the methods used to detect fungal RCD are the same techniques used to identify mammalian apoptosis with some adjustments due to the presence of the rigid cell wall, etc. It should be noted that apoptosis can be followed by secondary necrosis while a higher concentration/intensity/duration of the apoptogenic stimulus may instead lead to primary necrosis [181,290,291,292,293]. Grosfeld et al. [16] analyzed 133 reports of fungal cell death and established that 82 (62%) involved apoptosis, 11 (8%) involved necrosis and 40 (30%) involved both.

### 6.1. Cell Viability

A simple method of demonstrating cell viability is a survival assay, where the number of colony-forming units (CFUs) before and after treatment is counted and the percentage survival determined [294] (Appendix A). The addition of cyclohexamide or a caspase inhibitor (e.g., Z-DEVD-fmk) to the cells during culture can determine if loss of cell viability involves protein biosynthesis or caspase activity respectively [294]. Alternatively, cells may be subjected to serial dilution, spotted onto YPD agar and inspected after growth for 2 days at 28 °C [295] (Appendix A). This assay may be carried out after cell treatment in liquid culture, or the RCD-inducing substance may be incorporated into the agar. Another way to assess cell viability is to test the ability of cells to exclude certain dyes, including propidium iodide (PI), methylene blue, trypan blue and phloxine B [295] (Appendix A). However, viability assays do not distinguish between apoptosis and necrosis, and some viable cells cannot be cultured [296].

### 6.2. Cell Vitality

Other techniques assess vitality rather than viability, e.g., by measuring active transport or metabolization of a substance, such as FUN-1 (2-chloro-4-(2,3-dihydro-3-methyl-(benzo-1,3-thiazol-2-yl)-methylidene)-1-phenylquinolinium iodide), which is transported across the membrane of intact cells and then into the vacuole [295]. In the cytoplasm it appears as a diffuse green fluorescence but in the vacuole it forms red fluorescent cylinders. Vacuolar transport is a sign of cell vitality, but it should be noted that mutations in genes encoding components of the vacuolar transport machinery may block vacuolar transport and so vital cells may appear non-vital [297]. An alternative is to detect ATP, which is only generated in live cells. This can be carried out chemically, using firefly luciferase or one of many improved versions of the enzyme with different emission wavelengths/duration, etc., [298] or an ATP-binding, fluorescent protein may be expressed in yeast cells [299]. The bioluminescence protocol may be modified to incorporate cell culture in a 96-well microtiter plate and measurement of bioluminescence using a microplate reader (https://www.cellsignal.com/products/cellular-assay-kits/firefly/luciferase-atp-assay-kit/28854 Accessed on 20 May 2026). By combining ATP assay with other cell viability/vitality assays, Paciello [300] identified a subpopulation of cells they termed “resilient”. These cells survived the stress that killed other subpopulations but were unable to replicate. The QUEEN (quantitative evaluator of cellular energy) ATP biosensor is based on the epsilon subunit of *Bacillus subtilis* ATPase F_0_F_1_ fused to eGFP, an enhanced version of the green fluorescent protein sequence from *Chiridius poppei* [299,301]. The peak emission wavelength is 520 nm but the excitation peak is 410 nm and 480 nm for the ATP-bound and unbound forms respectively. The ratio of emission intensities for excitation at 410 nm and 480 nm is proportional to the local concentration of ATP. There are several versions of the QUEEN biosensor, including some that are targeted to different parts of the cell to measure ATP concentrations there. It is possible to use standard methods of plasmid amplification with PCR and transformation using lithium acetate, PEG and carrier DNA [302,303]. The selection marker is the *HIS3* gene for transformation of yeast strains with a histidine auxotrophy because they lack the *HIS3* gene. An alternative plasmid is available with a *URA3* marker for transformation of strains with uridine auxotrophy (https://yeast.nig.ac.jp/yeast/by/PlasmidDetail.jsf?id=9654; Accessed 20 May 2026).

### 6.3. Nucleus and DNA

The TUNEL assay is used to identify DNA strand breaks, which are hallmarks of mammalian apoptosis. It involves the use of terminal deoxynucleotidyl transferase to attach a deoxynucleotide, conjugated with a fluorescein, to the 3′ hydroxyl group at a DNA strand break [304] (Appendix A). TUNEL does not distinguish between single-stranded and double-stranded DNA breaks, and it was difficult to be sure that the dsDNA breaks, typical of mammalian apoptosis, also occurred in fungal cells undergoing RCD. Therefore, an alternative technique was developed, involving the use of small dsDNA probes, conjugated with fluorescein [305] (Appendix A). The probe only ligates to dsDNA breaks and Ribeiro and coworkers [305] showed that hydrogen peroxide- and acetic-acid-induced RCD in yeast does not involve double strand breaks, distinguishing these forms of yeast RCD from mammalian apoptosis. An alternative way to view DNA fragmentation is pulse field gel electrophoresis, where the electric field is pulsed in forward and reverse to better separate large fragments of DNA [305] (Appendix A). It was demonstrated that RCD-related DNA fragmentation involved active enzymes and that higher hydrogen peroxide concentrations that elicit necrotic rather than apoptotic cell death, do not lead to DNA fragmentation while lower, apoptogenic concentration do. The authors suggested that DNA fragmentation resulted from multiple ssDNA strand breaks rather than widespread dsDNA strand breaks. Therefore, fungal RCD differs from mammalian apoptosis in that dsDNA are a hallmark of the latter but not necessarily the former. Another method of detecting DNA breaks is to run DNA on a gel using electrophoresis but as the fragments may be large, it is necessary to use pulsed field gel electrophoresis, in which the direction of the current is continuously changing, improving the passage of large DNA fragments through the gel [305,306] (Appendix A). Mammalian apoptosis also involves fragmentation of the nucleus, and this may be visualized using DAPI, which binds at the minor groove of DNA and fluoresces blue [307]. As the nucleus breaks into multiple pieces, this phenomenon can be viewed with a fluorescence microscope [308]. However, DAPI is not membrane permeable and would normally only enter dead cells in which membrane integrity was already compromised. Therefore, it is necessary to permeabilize cells, e.g., with ethanol, which also fixes the cells (Appendix A).

### 6.4. ROS and Mitochondria

Methods for detecting ROS in yeast cells include the use of dihydroethidium (DHE), a cell-permeable dye that is oxidized by superoxide anions to yield ethidium, which fluoresces red [287,309] (Appendix A). Alternatively, the conversion of yellow nitro blue tetrazolium to blue formozan by superoxide anions or oxidation of hydrolyzed 2′,7′-dichlorodihydrofluorescein (H_2_DCF) to fluorescent green DCF by hydroxyl ions may be used to detect ROS in dying cells [304,310] (Appendix A). Methods for extracting cell contents to measure DCF levels include disruption of cells using glass beads, sonication and enzymatic digestion of the cell wall but these methods take time and a quicker method was developed, based on the use of lithium acetate to permeabilize cells [310] (Appendix A). Mitotracker orange (MTO) enters mitochondria and mitochondrial MTO fluorescence is proportional to mitochondrial membrane potential (MMP), so MTO can be used to assess MMP [311,312].

### 6.5. Plasma Membrane Changes

Annexin V labeling may be used to observe the flipping of phosphatidyl serine (PS) from the inner to the outer leaflet of the plasma membrane (which is typical of apoptosis in mammalian cells) and may be combined with PI staining [308] (Appendix A) to allow necrotic cells to be seen as well as cells undergoing RCD. However, it is necessary to digest the cell wall to see the membrane blebbing, which may introduce some artifactual changes. Electron microscopy [133,313] (Appendix A) may be used to visualize margination and condensation of the chromatin and blebbing of the plasma membrane but is time-consuming and requires digestion of the cell wall or specialist treatments that are even more time-consuming. Mitochondrial fragmentation, loss of membrane potential and accumulation of ROS have been observed in various forms of RCD [314]. Fusion proteins containing a fluorescent protein (e.g., GFP) fused to the presequence of a mitochondrion-targeted protein allow visualization of the mitochondria under a fluorescence microscope [294] (Appendix A). However, it should be noted that it may be necessary to use a codon-optimized gene sequence, especially when expressing it in a CUG clade fungus such as *Candida albicans* [315].

### 6.6. Problems with Standard Methods

The guidelines and recommendations on yeast cell death nomenclature suggested techniques for distinguishing among the different cellular mechanisms [316]. Cell death may be assessed using PI staining and identifying the percentage of cells that are “PI-positive” while viability can be measured using counts of colony-forming units (CFUs) or growth rate assays. A positive PI (or trypan blue) result could relate to primary or secondary necrosis, but annexin V staining (for PS flipping) can help to distinguish between the two. Annexin V staining also highlights apoptosis, but it should be noted that yeast cells need to be spheroplasted prior to use (possibly altering cell behavior) and that PS flipping also occurs during some forms of necrosis. Measuring CFUs fails to distinguish between dead and senescent cells. A reduced growth rate could indicate a change in the cell cycle rather than dying of cells. Yeast vitality may be assessed by observing the conversion of methylene blue to a colorless form, the export of red phloxine B or specific enzyme activities. Of course, some activities occur in dying cells and others are not necessary for life. The production of reactive oxygen species (ROS) is another characteristic of cell death and may be measured using e.g., dihydroethidium, 2,7-dichlorodihydrofluorescein diacetate, but different stains detect a different range of ROS.

Other techniques, recommended for confirming apoptosis, include DAPI staining and electron microscopy to visualize chromatin condensation, TUNEL staining to identify DNA strand breaks, western blot or immunofluorescence microscopy, etc., to detect loss of certain proteins from the mitochondrial intermembrane space and fluorescent probes that highlight a loss of mitochondrial membrane potential via changes in fluorescence or localization [316]. However, all these techniques detect changes that are not unique to apoptosis but may also occur during necrosis. One solution is to use a combination of annexin V and PI staining to reveal the status of different cells. When staining occurs with PI but not annexin V, cells are undergoing primary necrosis. Staining with annexin V but not PI indicates probable apoptosis. Staining with both annexin V and PI is a sign of secondary necrosis—necrosis that follows apoptosis as the cells lose membrane integrity. Finally, staining with neither PI nor annexin V would suggest that the cells are healthy.

Wloch-Salamon and Bem [317] reviewed three types of cell death in *S. cerevisiae* (apoptotic, autophagic and necrotic) and methods for identifying them in yeast cells. They also pointed out advantages and disadvantages of each method. For example, they state that the preparation technique can lead to false positives during TUNEL testing. Furthermore, annexin V staining relies upon spheroplasting of cells before preparation and this can lead to artifactual PS exposure. Tests for caspase activity (or rather, aspartase activity with specific P1-P4 preferences) may be complicated by unspecific substrate proteolysis. One way to distinguish between RCD and non-regulated necrosis is to determine whether new protein biosynthesis is necessary for the phenomena to occur (i.e., whether it is abolished in the presence of cycloheximide) (reviewed by [191]).

### 6.7. Problems with Testing for Protease Activity

Synthetic caspase substrates and inhibitors are unsuitable for assessing metacaspase activity in yeast as they produce a significant number of false positives [151,318,319]. Caspases cleave peptides after aspartate at the P1 position but metacaspases cut after arginine or lysine. Based on the autoprocessing and substrate cleavage preferences of various metacaspases, it was suggested that better fluorogenic substrates be used, such as z-VRPR-AMC and corresponding inhibitors such as z-VRPR-FMK [320,321,322]. A new generation of non-peptide metacaspase inhibitors has also been developed, particularly those that inhibit parasitic protist metacaspases with pro-growth/development roles [322,323]. There is some evidence of non-specific staining of a fraction of live cells by caspase FITC-VAD-fmk [319]. An alternative substrate D_2_Rh (Rhodamine 110 bound to two aspartate residues) was shown to be a reliable indicator of aspartase activity without non-specific staining of live cells. It is now known that metacaspases cleave after arginine and lysine, so metacaspase-specific cysteine protease substrates would be more appropriate, at least in forms of RCD where metacaspase activity has been shown to mediate cell death.

### 6.8. A New Generation of Caspase Inhibitors

**Figure 17:**
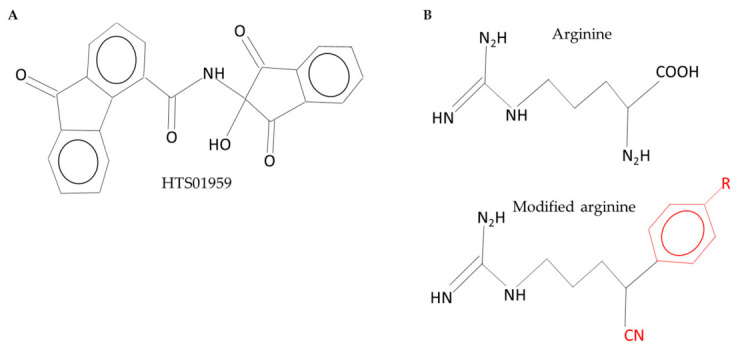
More specific metacaspase inhibitors. Metacaspase inhibitors have been developed that inhibit one or more parasite metacaspase and/or the growth of *T. cruzi* and *T. brucei* trypomastigotes (**A**). One set of inhibitors of variable efficacy was developed by amending the structure of arginine (**B**) since arginine is a favored P1 residue of metacapases. Modifications shown in red. Adapted from [322] (with permission from the American Society for Microbiology) and from [324].

The artificial metacaspase substrate Z-VRPR-AMC and inhibitor Z-VRPR-fmk have been tested on *T. brucei* MCA5 and yeast Mca1p. Since several plant and protist metacaspases have been shown to regulate development, there is considerable interest in using metacapase inhibitors as drugs [322]. Several putative inhibitors have shown promise, including HTS01959 (Figure 17A), which inhibited metacaspases in a dose-dependent fashion and reduced *Trypanosoma brucei* and *T. cruzi* trypomastigote numbers at a relatively low, non-toxic (to humans) concentration [322]. In another study, the structure of an arginine-based peptide was altered to place an electrophile in the P1 position (Figure 17B), leading to irreversible interaction with the catalytic cysteine residue of the metacaspase [324]. Some compounds were good inhibitors of *T. brucei* MCA2 and of the parasite itself but had no activity against caspase-3, indicating the absence of non-specific reactions.

## 7. Alternative Cell Death Proteases in Yeast

As discussed above, RCD resulting from various stresses is sometimes dependent on Mca1p and sometimes not. Additionally, deletion of *MCA1* may or may not affect the level of “caspase” activity detected in dying cells. The involvement of metacaspase-independent protease activity in RCD has been documented in several examples of RCD in *S. cerevisiae* [325]. It is possible that other proteases are responsible for the aspartyl protease activity that was detected during cell death in response to certain stimuli. In some experiments (above) a caspase inhibitor blocks cell death and/or aspartyl protease activity. Since there are no caspases in fungi, this may best be explained by a protease, with a catalytic cysteine, acting up- or down-stream of an aspartyl protease.

### 7.1. Esp1p

**Figure 18:**
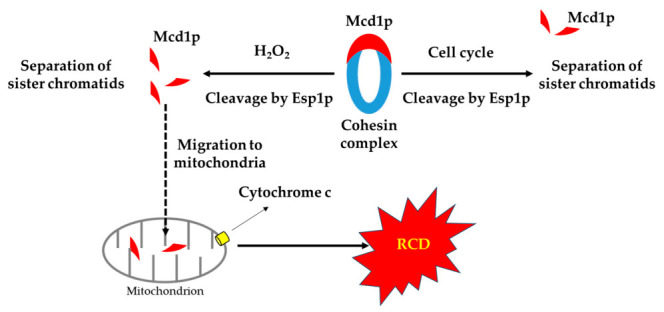
Involvement of cell death proteins, Esp1p and Mcd1p in the cell cycle. Mcd1p is a component of the cohesin complex that binds sister chromatids together. During the cell cycle, Mcd1p is cleaved by the separase Esp1p, allowing sister chromatids to separate. During treatment with apoptogenic concentrations of hydrogen peroxide, cleavage of Mcd1p again facilitates sister chromatid separation but the Mcd1p fragments migrate to the mitochondrion where they trigger loss of cytochrome c and regulated cell death. Adapted from [158].

For example, the yeast separin Esp1p is a clan CD protease with a catalytic cysteine and cleaves after glutamate and occasionally aspartate residues (MEROPS database, https://www.ebi.ac.uk/merops/ accessed 29 March 2026). *ESP1* is an essential gene with roles in mitosis and meiosis and cannot be deleted but overexpression leads to increased RCD (Saccharomyces Genome Database [SGD] https://www.yeastgenome.org, accessed 29 March 2026). During hydrogen peroxide-induced RCD, Esp1p is released from its inhibitor Pds1p and cleaves the cohesin complex component, Mcd1p, allowing the Mcd1p C-terminal fragment to migrate to the mitochondria and trigger cytochrome c-dependent RCD via loss of mitochondrial membrane potential [326]. It should be mentioned that Esp1p and Mcd1p are two of a number of proteins with roles in both cell death and the cell cycle [158]. During the cell cycle, Esp1p elicits the separation of Mcd1p from chromatin, allowing Mcd1p to promote the separation of sister chromatids (Figure 18).

### 7.2. Nma111p

Nma111p is a clan PA, S1 (chymotrypsin) family serine endopeptidase (MEROPS database, https://www.ebi.ac.uk/merops/ accessed 29 March 2026). Deletion of *NMA111* leads to loss of or reduced RCD while overexpression increases RCD (SGD https://www.yeastgenome.org/, accessed 29 March 2026). Nma111p, with roles in chromosome segregation and cytokinesis, is a homolog of the metazoan apoptosis-inducing mitochondrial protease Omi/HtrA2, and cleaves the inhibitor of apoptosis (IAP) protein, Bir1p (Figure 15) leading to RCD [327,328]. Deletion of *NMA111* reduces sensitivity of *S. cerevisiae* to sugar-induced cell death [329]. *C. albicans* cell death, induced by the *Lactiplantibacillus*-derived antimicrobial, SDZ3-1, led to ROS accumulation, inhibition of Glr1p expression and therefore reduced glutathione-dependent ROS scavenging, followed by metacaspase activation and RCD [330].

### 7.3. Kex1p

Defective N-glycosylation of proteins in a temperature-sensitive mutant was shown to induce cell death with typical apoptosis characteristics (DNA strand breaks, fragmentation of chromatin, PS flipping and ROS accumulation), accompanied by an increase in “caspase” activity (measured using a FITC-labeled pan-caspase substrate) and RCD was abrogated by the application of a pan-caspase inhibitor [170]. The use of different specific caspase substrates showed that N-glycolysation defect-dependent caspase activity was mainly directed towards substrates containing the caspase-6 target sequence VEID, followed by the caspase-8/10 target sequence, IETD, though cross-reactivity could not be ruled out. The serine carboxypeptidase, Kex1p mediated RCD, triggered by defective N-glycosylation, acetic acid and chronological aging and deletion of *KEX1* reduced markers of RCD and “caspase” activity in cells with defective N-glycosylation [331]. It should be noted that when tested in a cell-free experiment, Kex1p did not cleave the synthetic substrate containing the caspase-6 target sequence VEID, suggesting that the N-glycosylation defect-dependent caspase activity, mentioned above, might not be directly attributable to Kex1p. It has been shown that when yeast is killed by hypochlorous acid, produced by phagocytes, RCD and ROS accumulation is dependent on Kex1p [291]. Deletion of *KEX1* did not rescue *S. cerevisiae* from sugar-induced cell death, while deletion of *AIF1* or *NMA111* did [329].

### 7.4. Pep4p

The vacuolar aspartic protease, Pep4p was shown to migrate to the mitochondria during acetic-acid-induced RCD, where it mediated direct or indirect autophagy-independent degradation of damaged mitochondria, resulting in a reduction in cell death markers and enhancing viability [332]. Deleting *PEP4* was shown to stave off RCD while overexpression enhanced RCD. The role of Pep4p in mitochondrial degradation induced by apoptogenic levels of acetic acid was later shown to be dependent on its catalytic activity and on the ADP/ATP carrier Aac2p but not on the voltage-dependent channel Por1p [333]. Pep4p may be directly or indirectly responsible for at least some of the cell-death-related “caspase” activity, detected by various groups but Pep4p plays a pro-survival, rather than a pro-death, role in this example.

### 7.5. Proteasome

Using three fluorogenic artificial substrates, [334] demonstrated a 45%, 60% and 30% increase in proteasomal trypsin, chymotrypsin and peptidyl–glutamyl peptide bond hydrolyzing (PDPH) activity respectively during acetic-acid-induced RCD in yeast. It was shown that that proteasome inhibition blocked acetic-acid-induced cell death and that the increase in proteolytic activity was not due to an increase in proteasome component abundance. The authors speculated that activation/maturation/increased efficiency led to increased proteolysis. In *S. cerevisiae*, Nde1p passes electrons from reducing equivalents to the electron transport chain and is properly localized in the mitochondrion [335]. Defective mitochondria possess Nde1p that is partially exposed at the mitochondrial surface and the proteasome degrades Nde1p, yielding a toxic intermediate that triggers cell death. This may be a safety net, allowing for the removal of defective yeast cells from the population.

### 7.6. Other Candidate Death-Inducing Proteases

Fluorogenic caspase-1, caspase-6 and caspase-8 substrates were used to screen a library of yeast strains, deleted for protease genes [325] and they were further screened for susceptibility to acetic-acid-induced cell death. Six strains were found to have reduced caspase-1/6/8 activity and increased resistance to apoptogenic doses of acetic acid.

#### 7.6.1. Yps7p

Deleting *YPS7* reduced caspase-6 and caspase-8 activity and increased survival during acetic-acid-induced cell death. Yps7p (Yapsin 7 protein) is a putative GPI-anchored aspartic protease with a role in cell wall integrity (CWI) (SGD https://www.yeastgenome.org, accessed 29 March 2026). Deletion of *YPS7* might be expected to reduce resistance to acetic acid due to loss of CWI. However, it has been shown in *N. glabratus* that yapsins play a role in maintaining vacuolar pH [336] leading one to theorize that loss of Yps7 in *S. cerevisiae* might mediate RCD via the release of Pep4p from the vacuole.

#### 7.6.2. Aap1p

Deleting *AAP1* reduced caspase-6 activity and enhanced cell survival following treatment with apoptogenic doses of acetic acid. Aap1p is an alanine/arginine metalloaminopeptidase, involved in promoting glycogen accumulation during the diauxic shift, as well as positively regulating the heat shock response, both of which are pro-survival roles (SGD https://www.yeastgenome.org, accessed 29 March 2026).

#### 7.6.3. Pim1p

Deleting *PIM1* also reduced caspase-6 activity and enhanced survival of acetic acid-treated cells. Pim1p is an ATP-dependent Lon protease with roles in mitochondrial maintenance and respiration (SGD https://www.yeastgenome.org, accessed 29 March 2026). Interestingly, Pim1p is required for biosynthesis of mitochondrial intronic gene products, including cytochrome c oxidase subunit I (CoxIp). This suggests a possible link with cytochrome c, release of which drives some forms of RCD, and raises the possibility that loss of mitochondria and respiration (which promote ROS accumulation and therefore RCD) might be beneficial. In fact, expressing human α-synuclein in yeast leads to mitochondrially mediated cell death and yeast counteract this phenomenon by becoming rho^–^ petites, which lack mitochondria and respiration [337].

#### 7.6.4. Lap2p

The *LAP2* deletion mutant was defective in caspase-6 activity and was better able to survive acetic acid cell death conditions. Lap2p (leukotriene A4 hydrolase) is a leucyl aminopeptidase that also hydrolyses epoxide (SGD https://www.yeastgenome.org accessed 29 March 2026). The protein is involved in both protein catabolism and lipid metabolism and heat sensitivity is reduced in the deletant strain. Little is known about its involvement (if any) in cell death but if the protease mediates sensitivity to heat, it may promote sensitivity to other stresses and could be a pro-death protease under RCD-inducing conditions. Alternatively, it could be another pro-survival protein with indirect effects on aspartyl protease activity.

#### 7.6.5. Map1p

*MAP1* deletion reduces acetic-acid-induced cell death under apoptogenic conditions and has a deficiency in caspase-3 activity. Map1p is a methionine aminopeptidase that deletes N-terminal methionine residues of newly synthesized proteins and inhibits gene expression (SGD https://www.yeastgenome.org, accessed 29 March 2026). Deletion of *MAP1* has many detrimental effects including reduced RLS, cell size and survival under various stress conditions. Its localization includes stress granules, and it has been shown that the yeast metacaspase Mca1p has a role in clearance of protein aggregates, thus counteracting RCD by restoring proteostasis [280].

#### 7.6.6. Rbd2p

In the *RBD2* deletion mutant, caspase-3 and caspase-8 activity are depressed, relative to the wild type and the mutant strain is more resistant to acetic-acid-induced RCD (SGD https://www.yeastgenome.org, accessed 29 March 2026). Rbd2p is a putative clan ST rhomboid protease, and predicted membrane protein, that localizes to the COPI-coated vesicles, Golgi and nuclear periphery (SGD https://www.yeastgenome.org, accessed 29 March 2026; (MEROPS database, https://www.ebi.ac.uk/merops/ accessed 29 March 2026). In *S. pombe*, hypoxic conditions are believed to promote cleavage of the sterol regulatory element binding protein (SREBP) Sre1p, leading to migration of the N-terminal fragment (a transcription factor) to the nucleus, where it activates hypoxia response genes [338]. It was shown that Sre1p cleavage (and therefore growth under hypoxic conditions) was dependent on Rbd2p. Similar SREBP regulation of hypoxia genes also exists in *Aspergillus fumigatus* and *Cryptococcus neoformans*. If *S. cerevisiae* Rbd2p has a similar function, it is another pro-survival protein and its exact role in promoting acetic-acid-induced RCD remains to be established.

## 8. Defining Regulated Cell Death

### 8.1. Forms of Fungal Cell Death

The use of defunct terms such as apoptosis and apoptosis-like cell death to describe fungal RCD is frowned upon because the modern definition of apoptosis includes the involvement of caspase-3, which does not exist in fungi [339]. A group of fungal cell death researchers issued guidelines for the definition of different forms of cell death in yeast [316] and suggesting the use of specific terms. They made recommendations on how to distinguish between apoptosis, regulated necrosis, autophagy-dependent cell death (ADCD), accidental necrosis and “cytoprotective” autophagy. They also stressed that PCD is a specialized form of RCD, related to development (e.g., apoptosis of cells to sculpt fingers and toes). Apoptosis, regulated necrosis and ADCD are all forms of RCD while accidental necrosis (AN) is “unplanned” cell death, resulting from cellular insult, such as antifungal drugs, starvation, toxins and physical damage. General autophagy is a mechanism for recycling cellular material via the vacuole.

### 8.2. Similarities Between Fungal Cell Death and Mammalian Pyroptosis

Many researchers have compared fungal RCD with mammalian apoptosis but there are problems with such a comparison. The absence of true caspases, the less specific nature of metacaspase cleavage, the different mode of activation of metacaspases and the fact that fungal cytochrome c does not form a complex similar to the apoptosome after release form mitochondria all suggest that fungal RCD might be very different from mammalian apoptosis. Heterokaryon incompatibility (HI) in various fungi has been compared with mammalian systems for responding to non-self recognition [89]. HI defends individuals from viral infection and the theft of nutrients and HI is regulated by *het* genes, including NOD-like receptors (NLRs) that form a link between non-self recognition and cell death and are found in fungi, mammals and many other organisms. Other het genes are related to mammalian gasdermins or other pore-forming proteins that execute cell death via permeabilization of the cell membrane. These classes of pore-forming proteins are involved in mammalian pyroptosis and necroptosis.

## 9. Conclusions and Recommendations

At least some metacaspases appear to have roles in RCD but they are not caspases. They have different P1 substrate specificities and P1 specificity is defined in the word “caspase”. Most metacaspases are activated by calcium whereas caspases are activated via proximity of caspase proteins and interaction between them. Metacaspases appear to be less specialized than caspases as cell-death regulators and have pro-survival roles, including cell cycle regulation, growth, differentiation and proteostasis. Having said that, metacaspases are certainly structurally related to caspases and at least one substrate is conserved between humans and yeast. The substrate is cleaved at one specific residue (an aspartate) by caspase-3 but at four different residues (arginine or lysine) by MCII-Pa. However, it is believed that the ancestors of caspases and caspase orthologs resembled a metacaspase. It is logical to expect that metazoa, with more complex cell death programs, should have evolved caspases with much more specific cleavage targets.

The most important future aim is to use metacaspase-specific substrates and inhibitors to study fungal cell death and associated protease activity, to identify the degradomes of fungal metacaspases and to elucidate the regulatory pathways in fungal RCD. However, there are numerous examples of metacaspase-independent cell death in fungi, so it is also important to elucidate the exact mechanism of each example of fungal RCD and reliable metacaspase inhibitors could help to rule out metacaspase-dependent cell death. Other proteases should be studied for RCD involvement via deletion or mutaton of protease genes and comparisons of the resulting degradomes with those of the wild type. Typically, trypsin is used to degrade proteins prior to LC-MS/MS analysis but metacaspases (and some other candidate death proteases) cleave at arginase or lysine, so alternative methods of generating peptides of suitable size should be investigated, e.g., the combination of two or more proteases for in-gel digestion. Identifying the cleavage targets of death-related proteases would allow the downstream pathways of fungal RCD to be uncovered.

Finally, more consideration should be given to the possibility that fungal RCD is like other forms of cell death rather than apoptosis. Mutation of the cleavage residue in gasdermins of different fungi would better allow researchers to test whether a particular form of RCD is gasdermin-dependent. It may be that fungal RCD in response to a specific stressor is executed via multiple mechanisms and the agents of RCD may have both pro-survival and pro-cell death roles.

## Figures and Tables

**Table 1:** Identification of RCD in fungi.

Species	RCD Trigger	References
*Ophiostoma multiannulatum*	Unbalanced growth	[72]
*Neurospora crassa*	Unbalanced growth	[73,74]
*Aspergillus nidulans*	Unbalanced growth	[75]
*Podospora anserina*	Heterokaryon incompatibility	[76,77,78]
*Cochliobolus heterostrophus*	Heterokaryon incompatibility	[79,80,81,82]
*Cryphonectria parasitica*	Heterokaryon incompatibility	[83]
*Neurospora crassa*	Heterokaryon incompatibility	[84,85,86,87,88,89]
*Podospora anserina*	Ascospore abortion	[90,91,92,93]
*Neurospora* spp.	Ascospore abortion	[92,94,95,96,97,98,99]
*Schizosaccharomyces pombe*	Ascospore abortion	[92,100,101]
*Venturia inaequalis*	Ascospore abortion	[102]
* Fusarium verticillioides *	Ascospore abortion	[103,104,105,106]
* Bipolaris maydis *	Ascospore abortion	[92,107,108]
* Coniochaeta tetraspora *	Ascospore abortion	[109]
*Coprinopsis* spp.	Fruiting body development	[110,111]
* Agaricus bisporus *	Fruiting body development	[112,113,114]
*Psilocybe* spp., *Panaeolus* spp., *Stropharia rugosoannulata, Coprinellus domesticus, Candolleomyces candolleanus, Tremella mesenterica, Otidea onotica, Peziza ostracoderma*	Fruiting body development	[113]
*Saccharomyces cerevisiae*	Yeast killer toxin	[115,116,117,118,119]
*Saccharomyces cerevisiae*	Sugar	[120,121,122,123,124,125,126,127,128,129]
*Saccharomyces cerevisiae*	*cdc48^S565G^* mutant	[130]
*Saccharomyces cerevisiae*	Bax expression in yeast	[18,131,132,133,134,135]
*Saccharomyces cerevisiae*	Oxygen stress	[136,137,138,139,140]
*Saccharomyces cerevisiae*	Acetic acid	[141,142,143,144,145,146]
*Saccharomyces cerevisiae*	Plant defense compound osmotin	[147]
*Saccharomyces cerevisiae*	Aging	[148,149,150,151,152]
*Saccharomyces cerevisiae*	Pheromone	[20,153]
*Saccharomyces cerevisiae*	Sodium chloride	[154]
*Saccharomyces cerevisiae*	Defects in mRNA decapping	[155]
*Saccharomyces cerevisiae*	Aspirin	[156,157,158]
*Saccharomyces cerevisiae*	Hypochlorous acid (HOCl)	[159,160,161]
*Saccharomyces cerevisiae*	Defects in DNA replication initiation	[162,163,164]
*Saccharomyces cerevisiae*	Hyperosmotic stress	[165,166,167]
*Saccharomyces cerevisiae*	Reduced sister chromatid cohesion	[168,169]
*Saccharomyces cerevisiae*	N-glycosylation defect-induced ER stress	[170]
*Saccharomyces cerevisiae*	Copper or manganese	[171,172,173]
*Saccharomyces cerevisiae*	Formic acid	[174,175]
*Saccharomyces cerevisiae*	Palmitate-induced ER stress	[176,177]
*Saccharomyces cerevisiae*	Accumulation of Ras2p in mitochondria due to *WHI2*, *HXK2* or *SNF1* deletion	[178,179,180,181]
*Saccharomyces cerevisiae*	Lack of potassium	[129,182,183]
*Saccharomyces cerevisiae*	Lack of H2B K123 ubiquitination	[184,185,186]
*Saccharomyces cerevisiae*	Lead	[187,188]
*Saccharomyces cerevisiae*	Gefitinib (EGFR inhibitor)	[189]
*Saccharomyces cerevisiae*	Cisplatin	[190]
*Saccharomyces cerevisiae*	Heat shock (45 °C)	[191,192,193]
*Saccharomyces cerevisiae*	Anacardic acid	[194,195,196]
*Saccharomyces cerevisiae*	Cold plasma	[197,198]
*Saccharomyces cerevisiae*	Nickel oxide nanoparticles	[199,200,201,202]
*Saccharomyces cerevisiae*	Citral and geraniol	[203,204,205]
*Saccharomyces cerevisiae*	Expression of caspase-1 in yeast	[206,207]
*Saccharomyces cerevisiae*	Mito/autophagy defects due to deletion of *PIL1*	[208]
*Saccharomyces cerevisiae*	Synthetic antimicrobial peptides	[209]
*Saccharomyces cerevisiae*	Nano-plastic-induced oxidative stress	[210,211]
*Saccharomyces cerevisiae*	Silver nanoparticles	[212]
*Saccharomyces cerevisiae*	Cohesion dysfunction	[169]
*Saccharomyces cerevisiae*	Deletion of AP-3 components or downstream kinase	[213]
*Saccharomyces cerevisiae*	Enhanced mitochondrial DNA damage due to *HAP4* deletion	[139]
*Schizosaccharomyces pombe*	Bax/Bak expression	[214,215,216]
*Schizosaccharomyces pombe*	Deficiency in diacylglycerols	[217,218,219,220]
*Schizosaccharomyces pombe*	Replication stress	[221,222]
*Schizosaccharomyces pombe*	Inositol deprivation-induced ER stress	[223,224]
*Schizosaccharomyces pombe*	Chronological aging	[220,225]
*Candida albicans*	Hydrogen peroxide, acetic acid and amphotericin B	[226,227,228,229,230]
*Candida albicans*	Caspofungin	[231,232,233]
*Candida albicans biofilm*	Amphotericin B	[234,235]
*Candida albicans*	Farnesol	[236,237]
*Candida albicans*	Aureobasidin A	[238]
*Aspergillus fumigatus*	Stationary phase	[239]
*Aspergillus nidulans*	Sporulation	[240,241]
*Aspergillus nidulans* *Aspergillus fumigatus*	Farnesol	[236,242]
*Aspergillus flavus*	Essential oils	[243,244]
*Aspergillus fumigatus*	UPR/antifungal drugs	[245]
*Aspergillus flavus*	Perillaldehyde	[246]
*Aspergillus niger*	Carvacrol	[247]
*Aspergillus flavus*	Hexanal	[248]
*Cryptococcus neoformans*	Radiation	[249]
*Histoplasma capsulatum*	Radiation	[249]
*Colletotrichum trifolii*	Ras mutant under starvation UV light Hydrogen peroxide Heat shock Sodium chloride	[250]
*Colletotrichum gloeosporioides*	Bcl-2	[251]
*Colletotrichum gloeosporioides*	Magnolol	[252]
*Fusarium oxysporum, Colletotrichum graminicola.*	Killer toxin	[119,253]

## Data Availability

No new data were created or analyzed in this study.

## Abbreviations

The following abbreviations are used in this manuscript:

PCD	Programmed cell death
RCD	Regulated cell death
DAPI	4,6-diamidino-2-phenylindole-dihydrochloride
TUNEL	Terminal deoxynucleotidyl transferase d**U**TP **N**ick **E**nd **L**abeling
TdT	Terminal deoxynucleotidyl transferase
Br-dUTP	5-bromo-2′-deoxyuridine 5′-triphosphate
FITC	Fluorescein isothiocyanate
Caspase	Cysteine-dependent aspartyl protease
DED	Death effector domain
CARD	Caspase recruitment domain
DD	Death domain
Apaf-1	Apoptotic protease-activating factor 1
TNFR	Tumor necrosis factor receptor
TNF-α	Tumor necrosis factor alpha
TNFR1	Tumor necrosis factor receptor 1
FasL	Fas ligand
TL1A	Tumor necrosis factor-like cytokine 1A
DR3	Death receptor 3
DR4	Death receptor 4
DR5	Death receptor 5
DcR3	Decoy receptor 3
FADD	Fas-associated protein with death domain
FLICE	Fas-associated death domain protein interleukin-1β-converting enzyme
c-FLIP	Cellular FLICE inhibitory protein
DISC	Death-inducing signaling complex
PS	Phosphatidylserine
PM	Plasma membrane
Ab	Antibody
T cells	Thymus-derived lymphocytes
MOMP	Mitochondrial outer membrane permeability
SMAC	Second mitochondria-derived activator of caspases
WD40	Tryptophan-aspartic acid (W-D) domain
Bax	Bcl2 antagonist X protein
Bak	B cell lymphoma 2-associated X protein
Bcl2	B-cell lymphoma 2
Bcl2-X_L_	B-cell lymphoma extra large
ER	Endoplasmic reticulum
HOCl	Hypochlorous acid
AP-3	Adaptor protein 3
UPR	Unfolded protein response
HI	Heterokaryon incompatibility
rsk	Resistance to spore killer gene
dsRNA	Double stranded RNA
dsDNA	Double stranded DNA
ssDNA	Single stranded DNA
M-P1	M1 species protoxin
Gag Pol	Group antigen polymerase
PI	Propidium iodide
H_2_O_2_	Hydrogen peroxide
ROS	Reactive oxygen species
MAP	Mitogen-activated protein
Ras	Homologous to RAS proto-oncogene
cAMP	Cyclic adenosyl monophosphate
PKA	Protein kinase A
AIF	Apoptosis-inducing factor
AMID	AIF-like mitochondrion-associated inducer of death
IAP	Inhibitor of apoptosis proteins
TatD	Twin arginine translocation D
EGTA	Ethylene glycol-bis(β-aminoethyl ether)-N,N,N’,N’-tetraacetic acid

## Appendix A. Fungal RCD Assay Techniques

Survival assay [294]
Suspend yeast cells in sterile water (before and after treatment)
Calculate cell density and dilute to 10^−4^ cells/mL with sterile water
Spread on yeast extract peptone dextrose (YEPD) agar plates (1% yeast extract, 2% peptone, 2% glucose, 2% agar)
Incubate at 30 °C
Count colonies formed by the spread cells to determine number of colony forming units (CFUs)
Determine the percentage survival of cells: (post-treatment CFU/pre-treatment CFU) × 100

Serial dilution and spotting assay [295]
Suspend cells in sterile water
Dilute to cell densities (cells per mL) 10^7^, 10^6^, 10^5^, 10^4^
Drop 5 µL of each cell suspension onto YPD agar
Incubate at 28 °C for 2 days
Examine colony growth

Phloxine B and time-lapse photography [295]
Prepare a stock solution of 2 mg/mL phloxine B in sterile water and filter sterilize
Prepare cell suspension in 100 mM phosphate buffer (pH 7) containing 0.1% glucose, 1 mM EDTA and 10 µg/mL phloxine B
Spread 5 µL of this cell suspension on a YPD/agar plate
Incubate at 28 °C and capture images at various time points
Dead cells stain red, so it is possible to observe the gradual loss of viability throughout a cell population over time

Methylene blue [295]
Prepare methylene blue stock solution (0.1 mg/mL methylene blue in 2% aqueous dihydrate sodium citrate solution)
Add 100 µL methylene blue stock solution to 100 µL cell suspension
Incubate at room temperature for 5 min
Examine > 200 cells under a microscope to count dead (blue) cells

Combined fluorescent staining with PI and FDA [295]
Prepare FDA stock solution (1 mg/mL fluorescein diacetate in acetone)
Prepare stock solution of PI (1 mg/mL propidium iodide in water)
Suspend cells in PBS
Stain cells with 5 μg/mL PI and 10 μg/mL FDA
Incubate at room temperature in dark for 20 min
Propidium iodide stains dead cells red and FDA stains live cells green
Count > 400 cells to establish percentage stained with FDA or PI using fluorescence microscope

Luciferase-dependent detection of ATP [300]
Firefly luciferase emits bioluminescence and is dependent on ATP
Kits are available to carry out this assay, including the ViaLight^®^ Plus kit from Lonza Rockland Inc., Rockland, Maine, USA, which contains Bactolyse^®^ to digest the fungal cell wall and AMR^®^ (ATP Monitoring Reagents), containing luciferin and luciferase, which oxidizes the luciferin in the presence of ATP to yield light
Add 50 µL of Bactolyse^®^ to 100 µL of cell suspension in a Eppendorf tube
Mix vigorously and incubate at at room temperature for 20 min
Add 100 µL of AMR^®^ to each tube
Mix vigorously and incubate at room temperature
Take readings at 2 min and 4 min after addition of AMR^®^
Use a tube luminometer to measure luminescence
Use the average of the 2 readings

Microplate reader luciferase assay for ATP (https://wal.com/products/cellular-assay-kits/firefly/luciferase-atp-assay-kit/28854 Accessed on 20 May 2026)
Several kits are available for ATP assay using a microplate reader setup, including the Firefly Luciferase ATP Assay Kit from Cell Signaling Technology, Danvers, Massachusetts.
The microplate reader should have a luminometer setting
Thaw reaction buffer and bring to room temperature
Mix Firefly Luciferase Reaction Mixture with whole bottle of buffer
Mix gently
Pipet 100 µL of cell culture to each well of a white 96-well microtiter plate
Include control wells without cells
Treat and incubate cells as required (addition of reagents/heat treatment etc.)
Add 100 µL reaction mix to each well
Mix on orbital shaker for 2 min
Incubate at room temperature without shaking for 15 min
Measure luminescence using plate reader

QUEEN assay for ATP [299]
Use a strain that has been developed to express the QUEEN biosensor or amplify one of the plasmids (QUEEN-2m with a *HIS3* or *URA3* auxotrophic marker or one of the targeted versions mito-QUEEN-2m or SEC71TMD-QUEEN-2m from https://yeast.nig.ac.jp/yeast/ Accessed 2 May 2026) using PCR and transform into yeast using standard methods [337,338]
Streak yeast from frozen stock onto YPD agar (2% yeast extract, 1% peptone, 2% glucose, 1.5% agar) and incubate at 30 °C for 24 to 48 h
Use a sterile toothpick to add yeast cells to 2 mL of synthetic complete medium minus histidine or uridine (SC-H, SC-U—1% succinic acid, 0.6% sodium hydroxide, 0.5% ammonium sulfate, 0.17% yeast nitrogen base, 0.1085% dropout powder, appropriate amount of adenine, leucine and either histidine or uradine)
Incubate at 30 °C overnight with rotation at 40 rpm
Add 0.15 mL overnight culture to 2.85 mL SC-H/SC-U in a 16 mL tube
Incubate at 30 °C/40 rpm for ca. 3 h (to mid-log phase)
Dilute cell culture 1:10 with SC-H/SC-U and pipet 100 µL of cell culture into concavalin A-coated glass-bottomed dish
After 5 min, remove medium and wash three times with 300 µL medium
Check cell density using microscope and repeat from ten-fold dilution if not dense enough
Add 4–5 mL medium and incubate at 25 °C for 30 min
Use an inverted fluorescent microscope to observe cells and record red fluorescent and bright filed images for at least 100 cells

TUNEL assay ([304] and https://worldwide.promega.com/ accessed 15 May 2026)
Add cells to poly-L-lysine-coated glass slides and dry at 37 °C for 5 min
Fix with 4% performaldehyde for 20 min at 4 °C
Wash twice with phosphate-buffered saline (PBS)
Incubate for 5 min with 0.2% Triton-X-100 to permeabilize cells
Then, use a TUNEL assay kit, e.g., Promega DeadEnd^TM^ Fluorometric TUNEL system
Incubate with 100 μL equilibration buffer for 10 min
Add 50 μL of TdT reaction mix, cover cells with cover slips and incubate at 37 °C in a humidified chamber for 1 h N.B. keep cells in dark for subsequent steps
Place slides in 2 × saline/sodium citrate (SSC) buffer for 15 min to stop the reaction
Wash three times in PBS for 5 min each
Add mounting medium e.g., Vectashield^®^ which contains 4′,6-diamidino-2-phenylindole (DAPI) and stains nuclei blue
Use fluorescence microscopy to observe green TUNEL staining and blue nuclei

*In Situ* ligation [305]
Attach cells to poly-L-lysine-coated microscope slides (see TUNEL assay, above)
To fill in 5‘ or 3‘ overhangs, incubate cells at 27 °C for half an hour in 10 μL of a solution of 70 mM Tris-HCl (pH 7.5), 10 mM dithiothreitol (DTT), 70 mM magnesium chloride and 5U of the Klenow fragment in the presence or absence (respectively) of 2.5 mM deoxy NTPs
Wash twice in water
Probes are palindromic dsDNA consisting of 21 bp, including a fluorescein-conjugated dTTP
Ligate the probe by incubating the cells at room temperature in a humidified chamber in the dark for 16 h with 14 μg/mL probe and 100 U/mL T4 DNA ligase in a buffer consisting of 5 mM magnesium chloride, 0.1 mM DTT, 66 mM Tris-HCl (pH 7.5), 15% polyethylene glycol (PEG MW 8000) and 1 mM ATP
Wash twice with water and then with 100 mM Tris-HCl (pH 9) for 10 min each time
Observe fluorescence using a fluorescence microscope or assay using microplate reader etc.

Pulsed field gel electrophoresis [305]
Fix cells for 30 min with 3.7% (*v*/*v*) formaldehyde
Wash twice with 0.05 M ethyldiaminetetraacetic acid (EDTA) (pH 8)
Digest cell walls by suspending cells at 1.2 × 10^9^ cells/mL in 0.05 M plus 3 mg/mL zymolase 100T
Combine the cell suspension with an equal volume of 2% (wt/wt) low melting point agarose at 40 °C
Incubate overnight at 37 °C with 0.45 M EDTA (pH 8) plus 7.5% (*v*/*v*) 2-mercaptoethanol
Wash three times in 10 mM TE (Tris-HCl (pH 8) plus 1 mM EDTA (pH 8))
Incubate overnight at 50 °C in 0.5 mM EDTA, 10 mM Tris-HCl (pH 8), 1 mg/mL proteinase K and 1% (wt/wt) sodium-*N*-lauryl sarcosinate
Wash five times (30 min each) at room temperature in TE (pH 8)
Store at 4 °C until needed
Incubate at 37 °C for 30 min with 1 U/mL S1 nuclease, 200 U/mL nicking endonuclease and 200 U/mL DnaseI in reaction buffer
Run on electrophoresis gels at 12 °C and an angle of 120 ° in 0.5% Tris/borate/EDTA buffer with a voltage of 6 V/cm with switching times of 60 s and 90 s for 15 h and 7 h respectively
Stain gel for 45 min with 0.8% ethidium bromide solution
Destain for 20 min
Visualize under UV light

DAPI staining of nuclei ([308] and https://www.thermofisher.com/ accessed 16 May 2026)
Prepare a 5 mg/mL stock solution of DAPI in water or dimethylformamide
Dilute to 300 μM in PBS for working stock then to 300 nM in PBS final dilution, as needed
Resuspend cells in 70% (*v*/*v*) ethanol to fix and permeabilize them
Suspend cells in DAPI/PBS solution to stain them
Observe via fluorescence microscopy

Reactive oxygen species (ROS) assay using NBT [304]
Grow yeast cells at 32 °C for 15 min in presence of 50 μM/L light yellow nitro blue tetrazolium (NBT)
NBT is converted to dark blue formozan by superoxide anions
Treat cells with ethanol to fix them and air dry
Dissolve formozan in 1120 μL of dimethylsulfoxide plus 960 μL of 2 mol/L potassium hydroxide
Use a spectrophotometer to measure absorbance at 630 nm
Use cells treated with 100 μmol/L hydrogen peroxide as a positive control for RCD
To demonstrate mitochondrial involvement, make a stock of 1 mmol/L rotenone in DMSO
Rotenone inhibits complex I of the electron transport chain
Add rotenone to yeast culture with final concentration of 50 nmol/L

Using dihydroethidium to measure ROS [309]
Suspend 10^6^ cells in 250 μL 4 µM dihydroethidium (DHE) in PBS and incubate for 10 min in the absence of light at 30 °C
Visualize cells with a fluorescence microscope or measure fluorescence using a microplate reader or count fluorescent cells using flow cytometry or use FACS analysis

Using H_2_DCF-DA to measure ROS [310]
Hydrolyzed 2′,7′-dichlorodihydrofluorescein diacetate (H_2_DCF-DA) is cell-permeable and is deacetylated by esterases in the cell, after which various ROS (including hydroxyl radicals and hypochlorous acid) oxidize H_2_DCF to green, fluorescent DCF. Then carry out one of the following:

1. Resuspend yeast cells in 10 μM H_2_DCF-DA

Incubate for 30 min
Centrifuge at 6000 *g* for 10 min
Resuspend in PBS and visualize under fluorescence microscope or with a microplate reader

2. After H_2_DCF-DA treatment: resuspend in water and disrupt cells using glass beads; or resuspend in PBS and sonicate five times for 1 min each time, cooling on ice between sonications; or wash in 1.2 M sorbitol/50 mM EDTA/2% mercaptoethanol, centrifuge (6000 *g* for 10 min) resupend in same buffer with 25 U/mL lyticase, incubate with agitation for 30 min, incubate resulting spheroplasts in 10 μM H_2_DCF-DA for 30 min, centrifuge then resuspend spheroplasts in PBS and gently sonicate them

Assay extract/lysate fluorimetrically e.g., using a microplate reader or flow cytometry

3. After H_2_DCF-DA treatment, resuspend cells in 2 M lithium acetate to permeabilize the cells

Centrifuge and resuspend cells in 0.01% sodium dodecyl sulfate (SDS) plus a drop of chloroform
Incubate with shaking for 2 min to expel dye from cells
Centrifuge cells and assay lysate fluorimetrically

Change in mitochondrial membrane potential [308]
Incubate cells in the dark at 37 °C for 30 min with 2 μg/mL Rhodamine 123
Rho123 is a membrane permeable dye with a positive charge that builds up in the negatively charged mitochondrial matrix. It fluoresces green but leaks from the mitochondrion when the membrane potential is reduced
Wash cells twice with PBS
Observe under fluorescent microscope or use microplate reader to assay fluorescence (excitation at 485 nm and emission at 525 nm)

Detecting MMP changes with mitotracker orange [311,312]
Prepare storage stock of 1 mM mitotracker orange (MTO) in DMSO, dilute to working stock of 1 μM MTO with Edinburgh Minimal Medium (EMM: 50 × salt stock, 1000 × vitamin stock, 10,000 × mineral stock)
Centrifuge cells and resuspend in 1 mL EMM
Dilute working MTO stock solution to 200 nM with EMM
Incubate cells in 1 mL 200 nM MTO/EMM at 30 °C for half an hour
Wash 3 times in 1 mL EMM and resuspend cells in 1 mL EMM
Transfer 200 μL of cell suspension to 35 mm glass bottomed dish coated with lectin
Incubate at RT for 10 min
Wash to remove non-attached cells and add 200 μL
Visualize using fluorescence microscopy

Mitochondrial fragmentation [294]
Construct a plasmid expressing a fusion protein of green fluorescent protein and the mitochondrion-targeting presequence of a protein such as subunit 9 of the F_0_-ATPase from *Neurospora crassa*
Transform your chosen yeast strain with this plasmid
Visualize the mitochondria of treated and untreated cells under a fluorescence microscope
Identify any mitochondrial fragmentation that occurs in treated but not untreated cells

Annexin V-FITC/PI staining [308]
Use fluoroisothiocyanate-conjugated annexin V (annexin V-FITC) kit e.g., from Biovision
Wash cells with buffer containing 1.2 M sorbitol, 0.5 mM magnesium chloride and 35 mM dibasic potassium phosphate at pH 6.8
Incubate cells at 28 °C for 20 min in 15U/mL lyticase to digest cell wall
Wash twice and resuspend in binding buffer
Add 2 μL each of propidium iodide (PI) and annexin-FITC to 38 μL of cell suspension
Incubate at room temperature for 20 min
Observe via fluorescence microscopy or analyze via flow cytometry

Electron microscopy [313]
Freeze cells in a high-pressure freezer
Fix in phosphate-buffered glutaraldehyde
Digest the cell wall using lyticase (see annexin V staining above)
Embed in resin e.g., Epon^TM^ epoxy resin:
Wash with 100% ethanol
Wash with 100% acetone
Incubate for 30 min with 50% acetone/50% Epon
Incubate in 100% Epon for 20 h
Incubate further in fresh 100% Epon for 48 h at 56 °C
View via electron microscopy
Cut ultrathin section using an ultramicrotome
Stain with lead acetate
Visualize cells using electron microscopy

Caspase activity ([308] and https://www.merckmillipore.com/ accessed 15 May 2026)
Wash 5 × 10^6^ cells in PBS and resuspend in 200 μL FITC-VAD-fmk staining solution
Incubate at 30 °C for 20 min
Wash (1 mL) and resuspend (200 μL) in PBS
Incubate at room temperature for 10 min with 2 μg/mL PI
FITC-VAD-fmk is a cell-permeable, FITC-labeled version of the pan-caspase inhibitor carbobenzoxy-valyl-alanyl-aspartyl-[O-methyl]-fluoromethyl ketone (Z-VAD-fmk), which irreversibly binds to and inhibits cysteine proteases
Visualize via fluorescence microscopy or count stained cells via flow cytometry with excitation wavelength of 488 nm and emission wavelength of 525–550 nm
